# PAX: Using Pseudonymization and Anonymization to Protect Patients’ Identities and Data in the Healthcare System

**DOI:** 10.3390/ijerph16091490

**Published:** 2019-04-27

**Authors:** Mishall Al-Zubaidie, Zhongwei Zhang, Ji Zhang

**Affiliations:** 1 Thi-Qar University, Nasiriyah 64001, Iraq; 2 Faculty of Health, Engineering and Sciences, University of Southern Queensland, Toowoomba, QLD 4350, Australia; Zhongwei.Zhang@usq.edu.au (Z.Z.); Ji.Zhang@usq.edu.au (J.Z.)

**Keywords:** anonymity, ECDSA, electronic health record (EHR), PAX, pseudonym, XACML

## Abstract

Electronic health record (EHR) systems are extremely useful for managing patients’ data and are widely disseminated in the health sector. The main problem with these systems is how to maintain the privacy of sensitive patient information. Due to not fully protecting the records from unauthorised users, EHR systems fail to provide privacy for protected health information. Weak security measures also allow authorised users to exceed their specific privileges to access medical records. Thus, some of the systems are not a trustworthy source and are undesirable for patients and healthcare providers. Therefore, an authorisation system that provides privacy when accessing patients’ data is required to address these security issues. Specifically, security and privacy precautions should be raised for specific categories of users, doctor advisors, physician researchers, emergency doctors, and patients’ relatives. Presently, these users can break into the electronic systems and even violate patients’ privacy because of the privileges granted to them or the inadequate security and privacy mechanisms of these systems. To address the security and privacy problems associated with specific users, we develop the Pseudonymization and Anonymization with the XACML (PAX) modular system, which depends on client and server applications. It provides a security solution to the privacy issues and the problem of safe-access decisions for patients’ data in the EHR. The results of theoretical and experimental security analysis prove that PAX provides security features in preserving the privacy of healthcare users and is safe against known attacks.

## 1. Introduction

Data privacy is a prerequisite for any system, but especially for those systems, such as healthcare systems, that transmit user-sensitive data [1]. The healthcare system uses authorisation policies to enable healthcare providers to access required patients’ data. Ensuring patients’ privacy means preventing unauthorised users from accessing this data. Unfortunately, many healthcare systems transmit user requests or store policies with explicit plaintext, thus exposing patients’ data to the public. The personally controlled electronic health record (PCEHR) system provided by the National E-health Transition Authority (NEHTA) in Australia argues that security and privacy should be properly addressed in healthcare systems [2].

### 1.1. Security in EHR Systems

The security of medical records in the electronic health record (EHR) system has been a major focus of health and academic institutions, since the efficiency and quality of patients’ data management [1,3] by using the World Wide Web. EHR systems include identifications and patients’ data that require authorisation privileges to determine access control for authorised users [4]. Accurate medical data is essential for diagnosing diseases and determining the condition of patients during their online transfer from patient to healthcare provider [5,6]. Any change to this data causes health problems for patients. In addition, penetration of medical records of patients with diseases such as HIV infection or dermatological conditions can lead to discrimination, harassment, or even death of the patient if the diagnostic data changes during the transition from client to server [6,7]. In a broad sense, a terrorist may cause national instability by disclosing patients’ data, changing the data, destroying the data, or impersonating some patients [8]. Healthcare systems, and in particular, EHR systems, should provide end-to-end privacy for patients’ data. In addition, data storage and authorisation policies for patients in a central server yield data management gains but are an attractive target for hackers [8]. Therefore, there should be security mechanisms to protect the privacy of the patient as well as to prevent the penetration of policies on the server.

### 1.2. Privacy of Critical Medical Cases

The use of patients’ data for various purposes, such as consultations, access by a relative or caregiver, research, and emergency (secondary or indirect use) is a major challenge for authorisation systems; for example, the researcher should not exceed the limits of privacy granted to him/her [4]. In an emergency, when the patients’ doctor is unavailable or the patient does not have the capacity to give consent to another doctor, the patient’s privacy is seriously compromised [9]. In addition, if the patient is incapacitated, a relative is responsible for receiving the patient’s data [10]. Sometimes, the doctor also needs to consult another doctor to treat a patient’s condition. All these cases can result in the intrusion and penetration of data. The sharing of medical records among users of the EHR system allows patients’ data to be misused or abused by malicious breaches [11]. Many examples of penetration of the medical records for patients, such as medical staff who sold medical records to cancer patients, accessed medical records for patients at Washington University [12] or unauthorised access attacks exposed (June 2016) millions of healthcare records [13]. In 2018, the U.S. Department of Health and Human Services pointed out that unauthorised access/disclosure attacks targeted many health institutions and penetrated huge health records [14]. These penetrations show that the healthcare system requires a high level of security. Furthermore, an internal attack penetrates medical records more easily than external attacks because each practitioner has a privilege that allows him/her to access the server system. Many access control models have been used in the EHR, such as mandatory access control (MAC), discretionary access control (DAC), role-based access control (RBAC), and attribute-based access control (ABAC), and each model has specific authorisation mechanisms for data access [2]. In our project, we adopted the integration of the RBAC and ABAC to support a security level based on both role and user attributes. Therefore, EHR systems require mechanisms to ensure the privacy of patients’ data while protecting authorisation policies and healthcare provider requests [15]. In order to develop a successful project, privacy must be provided to the patient via the following measures:

1. Preventing attackers from accessing patient data and making data anonymous in case attackers do gain access to the data (i.e., external attacks).
2. Preventing legitimate users from exceeding their privileges (i.e., internal attacks).
3. Securing all requests, policies, and data of the change on the server or during the transfer between the clients and server to ensure the accuracy and reliability of patient data.
4. Applying anonymity to requests and policies to hide users’ identities.
5. Applying random pseudonym to requests, policies, and data to separate data associated with the real attributes of patients.

### 1.3. Our Contributions

Our contributions to providing full privacy and security of patients’ records can be summarised as follows:

- Combining ABAC and RBAC
  In this project, we integrate two existing models (ABAC and RBAC) to develop a system that provides handling of patients’ information at the coarse-grained and fine-grained levels. Our model fits the privacy and security requirements for medical records in the EHR by merging a user’s ID with the role as a single attribute entered in signature to identify subjects and objects.
- Separating users into two sets
  We have proposed separating users into direct and indirect sets for patients’ records to allow the server to distinguish between users’ requests. This significantly reduces the penetration rate of internal attacks.
- Using ECDSA’s signatures with XACML
  The anonymity property has been applied to the requests and policies of subjects. This feature was used during the implementation of the ECDSA signature algorithm with XACML to prevent attackers from determining the identity of healthcare providers (to prevent knowledge of the relation between a physician with a particular patient).
- Using Shamir scheme with signatures
  We used the Shamir scheme with the ECDSA signatures in the third protocol of authorising indirect users. This procedure is necessary to verify unauthorised users of patients’ data who could be conducting serious attacks on the EHR system.
- Using random pseudonym with patients’ data
  The pseudonym property has been applied to the requests and policies of subjects and resources. This feature prevents hackers from knowing that the data belongs to a particular patient (separating data from real attributes).
- Validating PAX scheme
  PAX scheme has simulated with an automated validation of Internet security protocols and applications (AVISPA) tool that is an efficient and flexible tool for testing and analysis attacks in modern research. AVISPA has used to validate that PAX is secure against both passive and active attacks. Additionally, Burrows, Abadi and Needham (BAN) logic has used to ensure request source, freshness and entity legitimacy.

### 1.4. Structure of the Paper

The report proceeds as follows. Section 2 discusses previous studies related to our research. Basic concepts about the techniques used in the PAX system will be introduced in Section 3. Section 4 describes the proposed authorisation model. Section 5 describes users’ scenarios and security analysis in the authorisation system. Section 6 presents comparison between PAX and previous studies. The conclusion and recommendations for future work are presented in Section 7.

## 2. Related Work

This Section discusses related works [2,6,8,9,16,17,18], and highlights their shortcomings.

The PERMIS project was proposed by [16] with the RBAC model. It described the conceptual authorisation of the credential validation service (CVS) before the approval stage of the access decisions for the resource as well as the distributed management of the credentials. However, the PERMIS system does not adequately protect the CVS. PERMIS also suffers from the problem of inheriting managers for all the attributes of their followers (hospital department managers or specialist doctors who inherit all their practitioners’ attributes and thus have access to patients’ data, which can lead to significant internal attacks) and also uses one signature of a public key cryptography (PKC#12) file for policies and attributes.

Quantin et al. [8] suggested using non-central medical records to eliminate issues of standardization and structure in data access requests. However, this scheme suffered from the use of a single aggregator that was similar to the dataset on the central server, which is vulnerable to attack. In addition, patients’ data comes from different sources and have different structures and standards; this difference causes a burden on the aggregator. Moreover, the authors used Rivest, Shamir, and Adleman’s (RSA) encryption algorithm, and this algorithm uses a large key size of 1024 bits, which causes a burden on the server. In addition, the aggregator needs time and storage to convert the data into a single context. Furthermore, this scheme suffered from the collision and doubloon problems due to the transference and transformation of patients’ data contexts.

The pseudonymization of information for privacy in an e-health (PIPE) project was designed to protect health data in the EHR through a layered system that included many keys such as an external key pair, an internal key pair, a symmetric key pair, and a shared key. It relied on RBAC to protect the keys [6]. This scheme used the Shamir scheme as a backup mechanism to retrieve the patients’ keys in the case of the loss of the smart card. However, this scheme did not explain the symmetric and asymmetric encryption algorithms used to generate pseudonym for users. In addition, the scheme increases the complexity of the server system with the use of many keys, especially if the scheme is used by a large health institution. In addition, the server must use the keystore to store the keys, and this requires protection and a storage space on the server.

Gajanayake et al. [2] integrated four access control models (DAC, MAC, RBAC, and purpose based access control [PBAC]) to obtain a single model that limits user access control of the medical record. However, their scheme addressed only the doctor and the patient and did not address different classes of healthcare providers. In addition, data and requests are clearly transmitted between client and server.

The healthcare system for patient privacy (HCPP) project was designed for the EHR to protect the privacy of patient data [9]. Researchers focused on an emergency scenario regarding the protection of patients’ data. They used a backup mechanism that allows the doctor to access patients’ health information without access to confidential parameters. However, this search relies on encrypting all patient data. When a client wants to access patient data, the server uses a keyword to perform an encrypted data mining operation. This process is very expensive for the server for two reasons. First, the server must encrypt the entire massive database with the continuous addition of new records, and second, the server must continuously mine each access request. In addition, their system did not support levels of authorisation and privileges (roles and attributes) that are more secure in providing privacy to patients’ records. In addition, researchers have reported that the patient has not been exposed to collusion because the patient does not attack himself, but this is not true because some impersonation attacks do the job without the theft or loss of the patient’s device. Moreover, this search did not specify the type of encryption algorithm used, which is very important for security and server performance, and addressed only emergency cases.

Jo & Chung [17] proposed an XML access control system (XACS) that enables users to access specific elements in an XML document. This system relies on removing certain parts of the XML document to allow users who are authorised to see certain parts of an XML document. However, requester information is transmitted explicitly over the Internet to a server, which makes it easier for an attacker to penetrate the privacy of users. In addition, it does not address internal attacks that are applied by legitimate users even though certain parts of the XML document have been removed.

Seol et al. [18] proposed an access control model based on partial encryption and XML signing in EHR’s documents within a cloud environment. Their model is supported in two phases: the first phase is access control using XACML and the second is to encrypt and sign data with XML. However, the cloud environment presents multiple security and privacy problems in the EHR system because of the distributed exchange of data between the various health centres. In addition, their scheme uses encryption in XML requests and responses, which will be extremely costly for legitimate entities exchanges in healthcare systems. In addition, in the first phase, requests and responses are clearly sent between the legitimate parties and therefore are exposed to attack. They also did not address the pseudonym mechanism that prevents access to real users’ information.

## 3. Overview of Security and Privacy Techniques in EHR Systems

**Figure 1:**
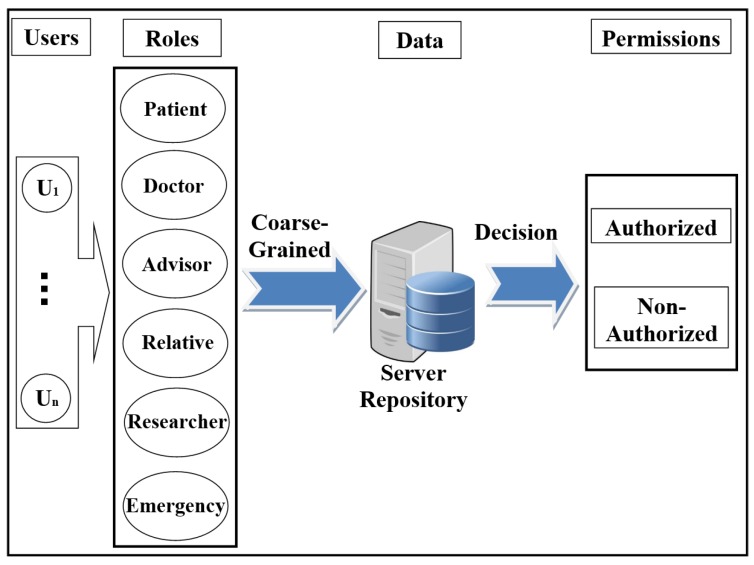
Scheme of role-based access control (RBAC) model.

**Figure 2:**
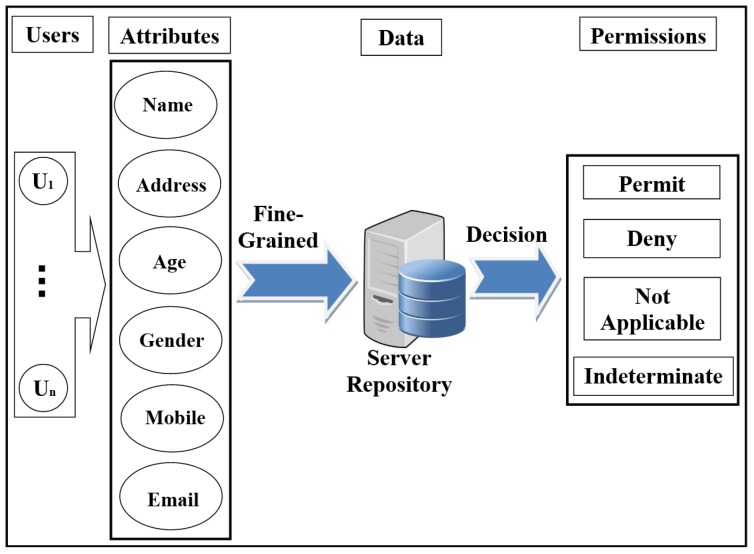
Scheme of attribute-based access control (ABAC) model.

**Figure 3:**
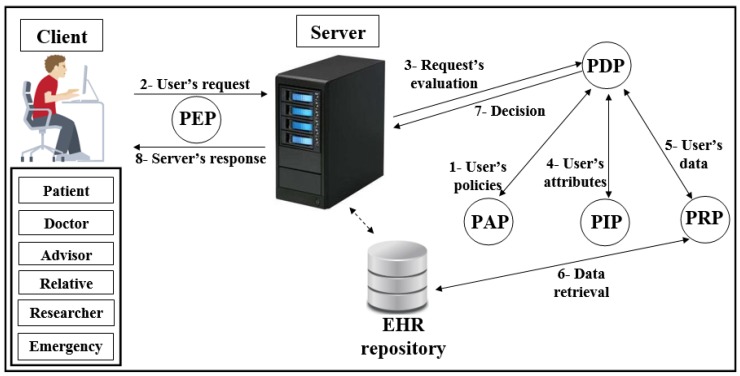
Scheme of XACML.

The EHR system needs a set of techniques to implement the management and privacy of patients’ data. In our project, we focus on the security aspect of authorising legitimate users. The EHR system collects and stores medical records on a server, and each medical record is associated with a set of attributes that allow healthcare providers or patients to access it later. Several countries, such as Australia, the USA, and the UK have implemented EHR by taking advantage of dealing with patients’ data over the Internet [5]. Therefore, our project used a set of techniques with the EHR. This section describes the threat model and the basic concepts of these techniques:

- Threat model
  Many serious risks to healthcare systems that require the building of a threat model to detect weaknesses in these systems. Dolev-Yao threat model [19] is used to test users’ authorisation in PAX. It is a formal model, a practical way of analyzing authorisation protocols in real environments. This model is very efficient in examining and analyzing various attacks. We assume that attacks can be internal, external, active, and passive. Additionally, we suppose that attributes server (AS) is trustworthy and safe against information repository penetration attacks. In this model, we address the following threats:
  - The attacker can flood the server with intensive authorisation requests, which is to stop the service from healthcare’s users and destroy the network.
  The attacker performs an attack to penetrate the repository on the central server, to access the patient’s data and reveal their identities.
  - The attacker performs a Man-in-the-middle (MITM) attack to modify the data and to become a legitimate user in the network.
  The attacker sends a fake authorisation request during the execution of a forgery/impersonation attack to gain access to patient data.
  - The attacker can launch an eavesdropping attack to obtain authorisation requests, and then perform an analysis of these requests to detect the correlation between data, information, and pseudonyms.
  - The attacker can execute timing attacks by using the time period to reveal user authorisation information.
- Access control in EHR systems
  Any system needs access control (AC) models to determine users’ access to the data repository. There are many AC models, and each one depends on a particular method and set of rules. One of the most distinct AC models is role-based access control (RBAC). This model relies on the classification of users into roles, and each role has privileges and rights regarding data access [2]. With RBAC, the security of the system is based on the structure of the system’s roles assigned to users [20]. Each role in the system is assigned according to the job of the user in the organization [21]. RBAC was introduced to solve problems with previous access models such as DAC. As shown in Figure 1, the RBAC model divides users into roles (such as a patient, doctor, and researcher).
  In recent years, there has been significant interest in using the attribute-based access control (ABAC) model for the protection of data privacy. This model is designed to access data more accurately (fine-grained) and securely. It handles user attributes (such as name, address, age, mobile, location, time) to allow users to access the server’s repository. ABAC proposed to go beyond the limitations in the rules and design of the most well-known control access models (DAC, MAC, and RBAC) [22,23]. ABAC is a rich model because it deals with a wide range of user attributes. ABAC supports administration, authorisation of context-aware, risk-intelligence, and scalability in various applications such as the Internet, IoT, Big Data, cloud computing, and VANET [24]. The attributes in ABAC are categorized into subject, object, action, and environment. As shown in Figure 2, each user has a set of attributes that allows him/her to access data in the server.
- Distributed AC implementation technology
  The most important component in the proposed EHR system is the EHR repository. The repositories contain data in various forms because these systems have difficulties dealing with different coordinates for data. Therefore, the use of extensible access control (XML) is suitable for the exchange of various data via the Internet. XML is a symbolic language and uses a simple and flexible method designed to describe, exchange, and manage data across the Internet.
  However, XML should support security and privacy mechanisms that provide different levels of protection of sensitive data in the whole or part of the XML document [17]. Access to data is a major challenge in big data management systems (EHR) that use different techniques. In addition, the exchange of information over the Internet has become essential and needs to achieve access authorisation, particularly in healthcare applications. Extensible access control markup language (XACML) standards include both access control (authorisation) and data management based on XML in the different systems [25]. Effectively, XACML offers features for data access and authorisation for the users at the fine-grained level, which is the most flexible and effective [26,27,28]. This technology is presented by the organization for the advancement of structured information standards (OASIS). This standard has many of the features that qualify it for use on the Internet, such as combining policy, combining algorithm, attribute, multiple subjects, policy distribution, implementation independency and obligations [23,28,29].
  This technique is based on the specific policies first and then on many modules such as policy enforcement point (PEP), policy decision point (PDP), policy administration point (PAP), policy information point (PIP), and policy retrieval point (PRP) to evaluate the request for access [4], as shown in Figure 3 (PEP sends and receives requests and accesses responses to the repository; PDP evaluates the decision; PAP creates policies based on users’ attributes; PIP retrieves users’ attributes; and PRP retrieves the users’ data from the repository). The result of the decision (permit, deny, not applicable, indeterminate) is sent to the subject via PEP [23].
- Elliptic curve digital signature algorithm (ECDSA)
  Proposed by Scott Vanstone in 1992 [30], the elliptic curve digital signature algorithm (ECDSA) is an asymmetric signature algorithm that depends on the use of the points on the curve to sign data. It has been used to provide integrity, authentication, and non-repudiation properties in the communications network with limited capacity in terms of power and processing. The algorithm depends on the elliptic curve discrete logarithm problem (ECDLP). It is impervious against different attacks when the parameters are accurately selected [31], i.e., it is difficult to obtain *k* from *P* and *Q* (where *k* is an integer and *P* and *Q* are two points on the curve) [32,33]. ECDSA uses small parameters which expedites the performance of computations, thus reducing time and storage [34]. These features are very important for large organizations and constrained-source devices such as wireless sensor networks (WSN) that require processing power, memory, bandwidth, or power consumption [35]. More details about ECDSA’s signature and verification are available in [31].
- Shamir scheme
  The secret sharing scheme or the Shamir ($SS_{s}$, *t*) scheme depends on a set of keys/secrets sharing ($SS_{s}$) and threshold (*t*) to produce a master key/secret (MS). The master secret can be created from some or all of the $SS_{s}$ [36]. In this scheme, *t* specifies the minimum number of keys/secrets that allow reconfiguring MS [37,38]. This scheme consists of two phases: Generation and Reconstruction. In the Generation phase, the server divides MS into a set of secrets sharing ($SS_{1}$, $SS_{2}$, .., $SS_{n}$), and each client ($C_{i}$) securely receives one secret sharing (SS) that is part of MS. In the Reconstruction phase, $C_{i}$ needs to achieve any set of secrets ($SS_{s}$) required by relying on the value of *t* to construct MS (correctness and homomorphism properties). If $C_{i}$ has *t*-1 from $SS_{s}$, $C_{i}$ fails to obtain information from server (secrecy property). Calculating the MS is a very difficult operation for the attacker. In addition, the secrets that are configured for the MS are anonymous users; the attacker does not know if these secrets belong to any of the users [6]. The Shamir scheme provides an anonymity solution to generate a MS with several features such as full security in hiding $C_{i}$s’ $SS_{s}$, a MS size equal to $C_{i}$s’ $SS_{s}$ sizes, easy creation of a MS from a set of keys/secrets, and creation of a new key/secret for one-time use [33].

## 4. Our Proposed Authorisation Model

In this Section, we will provide details about our new authorisation scheme that support security and privacy mechanisms to ensure legitimate users’ authorisation in healthcare applications. This Section will be divided into the network model, applying privacy concepts and PAX authorisation protocols for users.

### 4.1. Network Model

**Figure 4:**
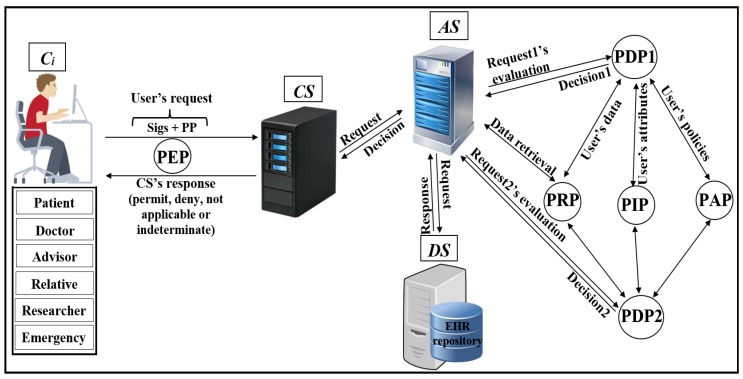
Pseudonymization and Anonymization with the XACML (PAX) model.

As shown in Figure 4, Pseudonymization and Anonymization with the XACML (PAX) is an authorisation system that works with EHR. The network model consists of four entities: client ($C_{i}$), central server (CS), attributes server (AS) and data server (DS). These entities communicate with each other in the PAX framework to accomplish authorisation and privacy preservation of users in access to the patients’ datasets. CS is the portal that prevents users from accessing directly to both AS and DS. Patients’ data are stored on the data server (DS) and are fully separated from the attributes of the users (patients and healthcare providers) that stored on the attributes server (AS). Each $C_{i}$ creates an access request and sends it to the CS. Then, CS verifies the authorisation information for the user’s request, if this request is valid, CS sends the authorisation request to AS for an evaluation; otherwise, CS sends the “deny” response to $C_{i}$. When AS receives the authorisation request from CS, AS evaluates the access request by PDPs modules, verifies signatures, pseudonyms, and other security parameters. If all evaluations and tests are valid, AS sends a request to DS to retrieve patient data; otherwise, AS sends the “deny” response to CS. After that, DS checks for signatures (Sigs) and privacy parameters (PP), if all operations are correctly performed, DS sends the required data with pseudonyms and Sigs to AS which in turn sends the “permit” response to $C_{i}$ by CS to allow access to the dataset. The authorised user will receive the “permit” response and the copy of the required data. The PAX system uses two PDPs (PDP1 and PDP2) to implement the user authorisation process, as shown in Figure 4. In this project, we focus on securing requests and policies to provide a high level of user privacy. PAX depends on the Balana Project, which is the only open source project that implements XACML v3.0 to ensure privacy and security for patients’ medical records.

### 4.2. Implementation of PAX

In this section, we will introduce the privacy concepts in PAX.

**Figure 5:**
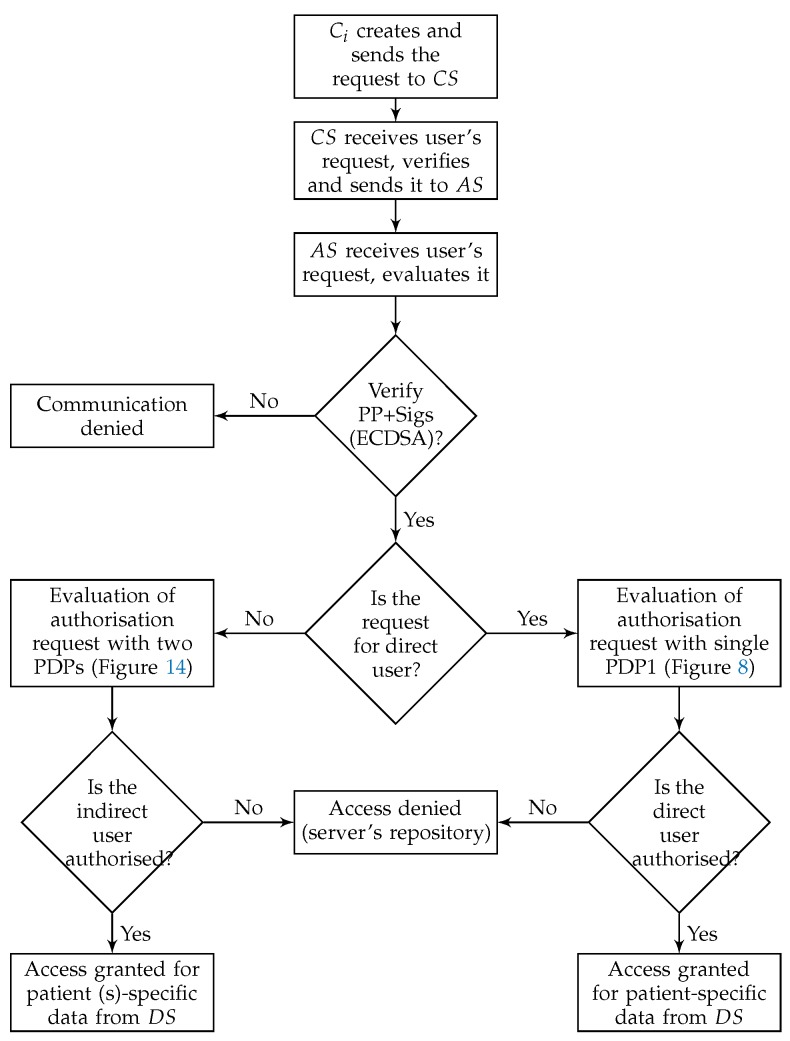
Authorisation of direct and indirect users.

**Figure 6:**
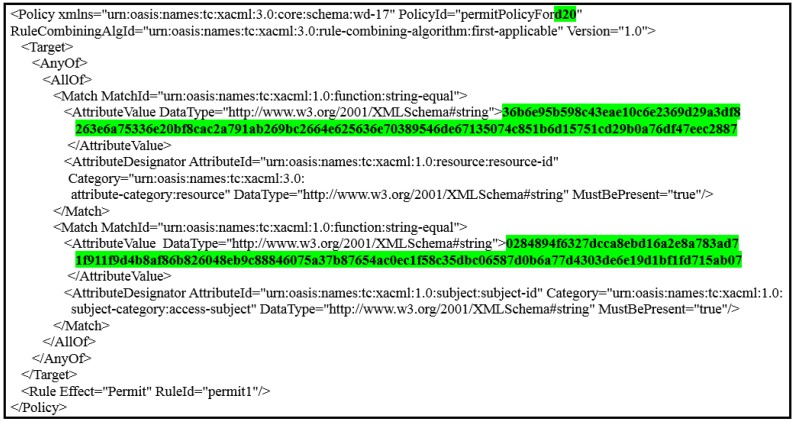
PAX policy.

**Figure 7:**
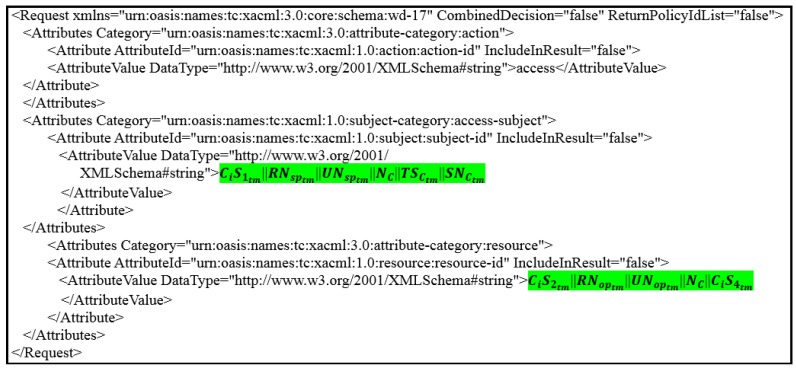
$C_{i}$’s request.

- **EHR’s users in PAX**
  Security and privacy address where, when, and why data is available and who can access the data repository. Patients and healthcare providers require services that are efficient, fast, and continuous and at the same time incorporate strict restrictions to determine data access. Therefore, AC to medical records has several challenges in terms of security and privacy:
  1. Legitimate users should not exceed their privileges.
  2. Users’ roles in the EHR system should be defined. For example, a doctor can have several roles, such as an emergency doctor and a researcher doctor.
  3. Data should be anonymous when it reaches the wrong user due to misuse or attacks.
  4. Compliance with medical standards for EHR (such as HIPAA) is essential.
  In PAX, we divide users into two categories:
  - Direct users: These users include those who are directly associated with the data, such as the patient and the doctor.
  - Indirect users: These users include those who are not directly and continuously associated with the data, such as advisors, patients’ relatives, researchers, and emergency doctors.
  Although PAX includes both categories of users, this project focuses on indirect users (Figure 5 shows a flow chart for authorisation of direct and indirect users in PAX). Any healthcare system can be exposed to an internal attack by indirect users if there are no security and privacy mechanisms to prevent them.
- **Users’ pseudonym in PAX**
  Several methods are used to protect the privacy of patients’ data, such as encryption and anonymization. However, these methods suffer from disadvantages. For example, encryption of patients’ data [7] has the following disadvantages:
  1. The researcher or emergency doctor will not benefit from the encrypted data, and if he/she can decrypt the patients’ data, this is a breach of security in the healthcare system.
  2. Large database encryption is very expensive for the server system, which leads to unnecessary time consumption and reduced processor performance [39].
  3. The database of patients’ data requires the continuous addition and deletion of records, and if the data is encrypted, this will increase the burden on the server [40,41].
  4. Encryption can contain direct information about the patients. The penetration of this encryption will leave the patients’ identity and information exposed [42].
  The anonymization of patients’ data requires the following:
  1. The removal of all the attributes associated with the patient that prevents the healthcare provider from dealing with the associated patient’s data [7].
  2. Adding a large set of counterfeit records, which greatly increases the size of the database and therefore consumes server resources, especially with the continuous use of the database by healthcare providers.
  To solve these problems, we apply random pseudonyms with PAX to separate the association between patients’ attributes and data. The medical records transmitted between the client and server do not contain any patients’ attributes. This prevents the attackers from identifying patients. In PAX, we propose to use four datasets: the first was for users’ attributes (patients and healthcare providers); the second was for applying pseudonyms to users; the third was for users’ policies (on AS); and the fourth was for patients’ data (on DS). When the EHR system wants to add a new healthcare provider or patient, the PAX randomly generates a pseudonym for that user and adds it to the second dataset. Suppose that we have a dataset for random pseudonyms, as in Table 1. PAX generates pseudonyms (such as p429 or d761) for patients or healthcare providers during the addition of a letter representing the user’s role (UR) such as *p* or *d* plus a random client’s number (CN). Each subject’s pseudonym (SP) and object’s pseudonym (OP) consists of UR and CN (internal pseudonym), which are not transferred between entities and are used for policy verification at AS. XACML’s request in PAX depends on the SP and OP (external pseudonym), and both SP and OP are divided into role’s number (RN) and user’s number (UN) (after replacing UR with RN and CN with RN) and the latter are segmented into three parts (low (l), medium (m), and high (h)) with length 8 bits per part as in Table 2. These pseudonyms are associated with the users’ IDs. It enables users to access a specific patient’s data without exceeding granted privileges and rights.
- **Using ECDSA’s signatures**
  PAX uses ECDSA (NIST prime-256) with requests and policies to ensure that security requirements apply to the privacy of patients’ data. We have applied ECDSA signatures with subjects’ and objects’ attributes to ensure integrity property to prevent changing attributes in requests and policies, authentication property to prevent external attackers and non-repudiation property to prevent authorised users from denying their requests to receive medical records. The application of security requirements is very important in systems that use sensitive data, such as healthcare systems. In PAX, the $C_{i}$ signs the request with pseudonyms (RN and UN), and the servers (CS and AS) verify the request’s Sigs. If valid, the AS assigns the request to the PDPs engines (after replacing Sigs(external pseudonym) with Sigs(internal pseudonym)) in XACML v3.0; otherwise, the request is rejected. PAX uses ECDSA’s Sigs to hide parts of SP and OP when exchanging XACML’s requests between PAX entities. The high performance and security level makes this algorithm suitable for application in large systems (such as EHR).
- **Policies administration in PAX**
  System Administrator is responsible for creating policies for healthcare providers and patients in AS by PAP. Policy in PAX consists of the policy ID, subject, object, and rules for policy implementation. The first process in the PAX system is to create datasets for pseudonyms and attributes for all users. The process of creating policies depends on previous datasets. PAX uses ECDSA to generate a signature of SP ($S_{sp}$) and a signature of OP ($S_{op}$) based on the pseudonyms (UR and CN) for both SP and OP. Creating signature-based policies and pseudonyms protects policies on the server in a way that is immune to internal and external attacks (policies do not depend on users’ real attributes). For example, the system administrator creates a user policy by entering the doctor’s name and UR and patient’s name, PAX creates this policy as shown in Figure 6. The policy parameters are highlighted in green: d20 represents the SP and uses as policy’s ID; the first long 128-bit hexadecimal number represents the $S_{op}$; and the second long 128-bit hexadecimal number represents the $S_{sp}$. This policy can include a set of rules such as determining the date of data access, the time specified on a given day, or the number of access times.
- **Clients’ requests and server’s responses**
  PAX’s users must create an authorisation request to access medical records. This request consists of subjects’ and objects’ attributes. The $C_{i}$ application in PAX uses the parts of RN and UN as a single attribute to generate the ECDSA’s Sig for the subjects and the objects. Figure 7 shows the client’s request to access patient data (where the request parameters are highlighted in green; $C_{i}S_{2_{tm}}||RN_{op_{tm}}||UN_{op_{tm}}\left|\right|N_{C}\left|\right|C_{i}S_{4_{tm}}$ in resource segment represents the object’s attributes; and the $C_{i}S_{1_{tm}}||RN_{sp_{tm}}||UN_{sp_{tm}}\left|\right|N_{C}$$||TS_{C_{tm}}\left|\right|SN_{C_{tm}}$ in access-subject segment represents the subject’s attributes). In addition, the $C_{i}$ application uses a part of $RN_{sp}$ to explain to the AS the user’s role to determine the desired policy after verifying the Sigs. Then, the $C_{i}$ sends the request to the AS by CS for evaluation. The AS evaluates the request in the PDP engines, and the response (permit or deny) returns to the $C_{i}$ by CS.
- **Using Shamir scheme**
  In PAX, we implemented the Shamir scheme to increase the level of security for indirect users (advisors, patients’ relatives, researchers, and emergency). Indirect users are legitimate users who can perform an internal attack because of the rights granted to them. PAX uses ECDSA to sign all signatures of healthcare users to create a master signature (MS). Then, PAX uses the Shamir scheme to generate secrets sharing ($SS_{s}$) from a MS. Each indirect user receives SS via a secure communication channel. $C_{i}$ needs a set of $SS_{s}$ to reproduce MS. PAX uses *t* = 3, which means that the randomly selected $SS_{s}$ require at least 3 $SS_{s}$ to generate MS. In addition, depending on $RN_{sp}$, AS specifies that the user’s role is indirect and use the Shamir scheme with ECDSA’s Sig to verify the original MS and then evaluate the request by PDP2. Using Shamir’s scheme with XACML adds the property of authenticity, as an indirect user cannot access data with the same $SS_{s}$. This operation enables PAX to secure the privacy of patients’ data and protect patients’ data from internal and external attacks. When an indirect user wants access to medical records, he/she does not know whether the $SS_{s}$ used to generate the MS belong to any specific healthcare providers.

### 4.3. PAX Authorisation Protocols

In this section, we will provide in detail PAX’s protocols framework in authorising direct and indirect users. PAX uses four protocols for direct users such as doctor and five protocols for indirect users such as researcher to secure communication among PAX’s entities. The request in protocols includes PP for a subject (sender) and object (receiver).

**Figure 8:**
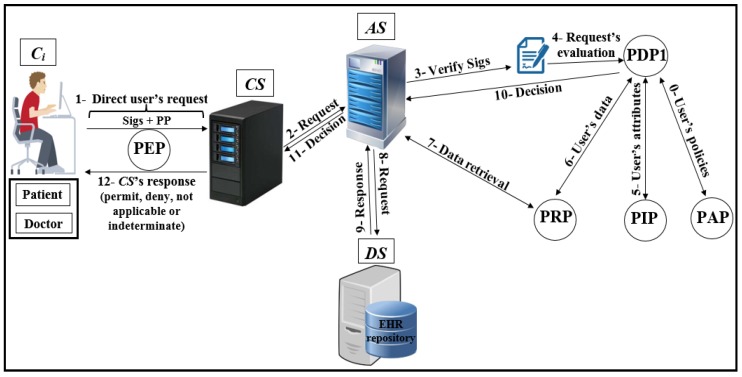
Authorisation of direct users.

**Figure 9:**
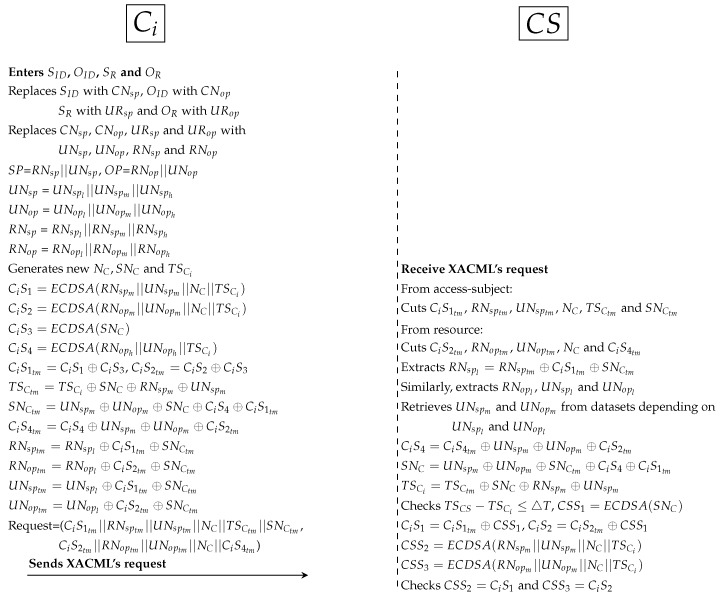
Protocol of PAX model between $C_{i}$ and CS.

**Figure 10:**
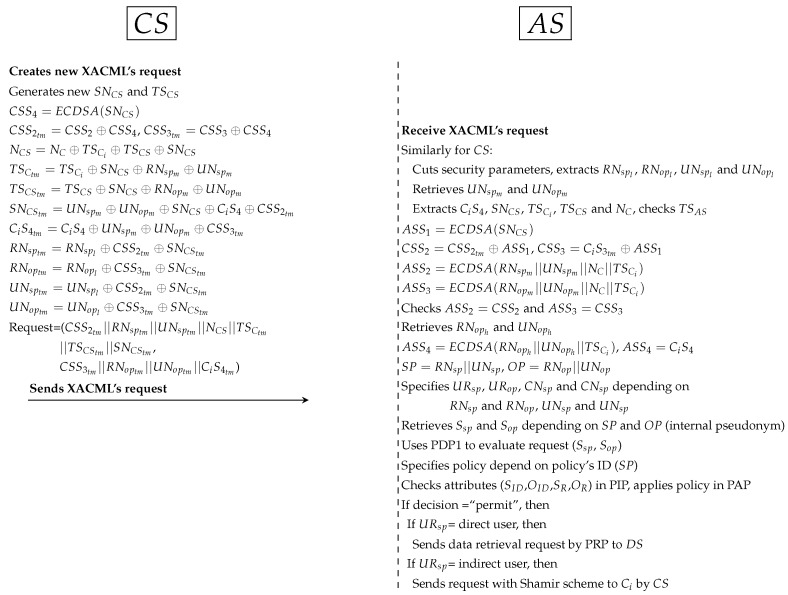
Protocol of PAX model between CS and AS.

**Figure 11:**
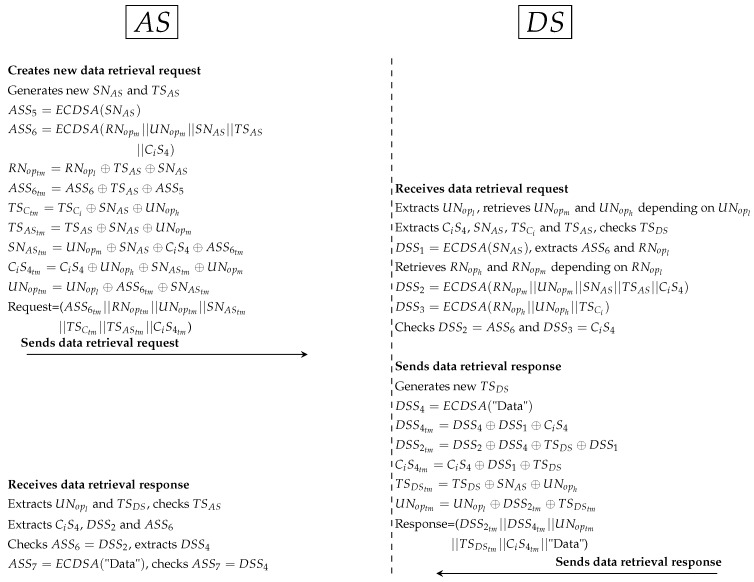
Protocol of PAX model between AS and DS.

**Figure 12:**
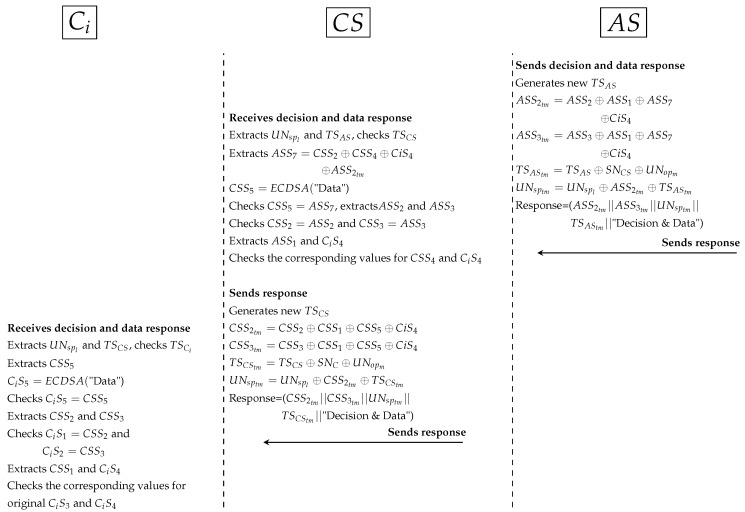
Protocol of PAX model between AS, CS and $C_{i}$.

**Figure 13:**
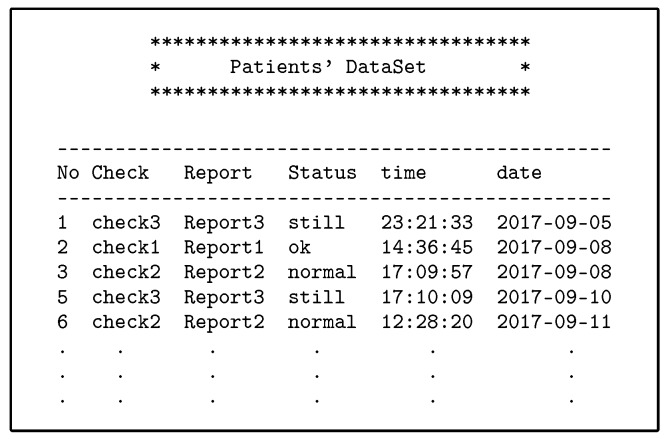
Part of medical records for patients.

**Figure 14:**
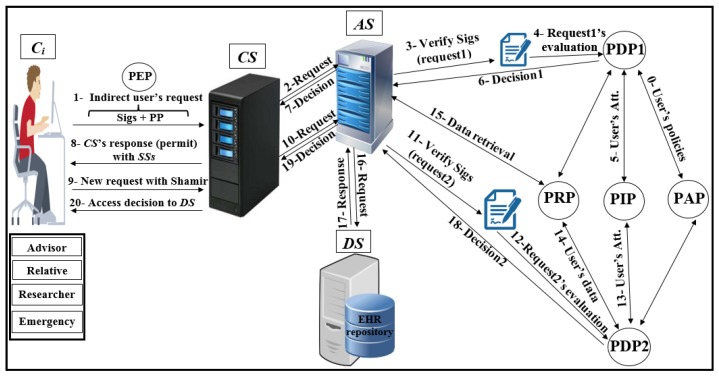
Authorisation of indirect users.

**Figure 15:**
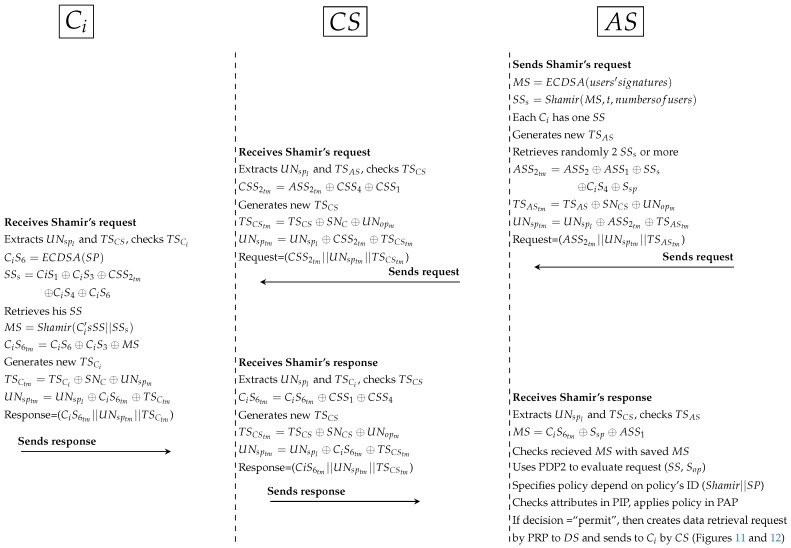
Protocol of PAX model for indirect users.

- **Authorisation protocols for direct subjects and objects**
  To run through the authorisation process for direct users of PAX, the security techniques mentioned in the previous sections will be the basis for building the PAX authorisation system. In this section, we will explain the protocols of authorising direct users such as doctors and patients to access medical records (EHR).
  - **Prerequisite procedures**
    There are a set of steps that must be taken before authorisation can begin.
    1. Create two datasets (attributes, pseudonym) on AS. If datasets are established, the processes are to add new users or delete direct users.
    2. Create policies (dataset 3) for all direct users based on anonymity and pseudonym.
    3. Storage of medical records (dataset 4) for patients in the DS’s repository (after collecting them from patients using wireless medical devices, this process requires security mechanisms, but the process of storing medical records safely is beyond the scope of this research). We assume that patients’ data is located on the DS.
  - **Authorisation protocols**
    The following protocols detail how the direct user is associated with the EHR in DS. Figure 8 depicts generally the authorisation process, while Figure 9, Figure 10, Figure 11 and Figure 12 show the authorisation protocols of direct users with PAX entities.
    1. First protocol as shown in Figure 9:
      * PAX’s user enters the subject ID ($S_{ID}$), object ID ($O_{ID}$), subject role ($S_{R}$) and object role ($O_{R}$) to the $C_{i}$ application. $C_{i}$ replaces $S_{ID}$, $O_{ID}$, $S_{R}$ and $O_{R}$ with $CN_{sp}$, $CN_{op}$, $UR_{sp}$ and $UR_{op}$ respectively. After that, internal pseudonyms are replaced with $UN_{sp}$, $UN_{op}$, $RN_{sp}$ $RN_{op}$ respectively. Then, $C_{i}$ generates random nonces ($N_{C}$ and $SN_{C}$) and new timestamp ($TS_{C_{i}}$). $SN_{C}$ is a random secret between $C_{i}$ and CS. $C_{i}$ computes 4 Sigs ($C_{i}S_{1}$, $C_{i}S_{2}$, $C_{i}S_{3}$ and $C_{i}S_{4}$). $C_{i}S_{1}$ and $C_{i}S_{2}$ is used to ensure the legitimacy of $C_{i}$ in CS. $C_{i}S_{3}$ is used to protect $SN_{C}$ between $C_{i}$ and CS. $C_{i}S_{4}$ is used to validate $C_{i}$ in both AS and DS (depending on $RN_{op_{h}}$ and $UN_{op_{h}}$). $C_{i}$ hides all Sigs such as $C_{i}S_{1}$ temporary ($C_{i}S_{1_{tm}}$) and PP such as $TS_{C_{tm}}$ and $SN_{C_{tm}}$. At this point, $C_{i}$ sends XACML’s request to CS that including subject’s information ($C_{i}S_{1_{tm}}||RN_{sp_{tm}}||UN_{sp_{tm}}\left|\right|N_{C}$$||TS_{C_{tm}}\left|\right|$$SN_{C_{tm}}$) and object’s information ($C_{i}S_{2_{tm}}\left|\right|$$RN_{op_{tm}}||UN_{op_{tm}}\left|\right|N_{C}\left|\right|C_{i}S_{4_{tm}}$).
      * CS receives XACML’s request from $C_{i}$, cuts Sigs and PP from access-subject ($C_{i}S_{1_{tm}}$, $RN_{sp_{tm}}$, $UN_{sp_{tm}}$, $N_{C}$, $TS_{C_{tm}}$ and $SN_{C_{tm}}$) and resource ($C_{i}S_{2_{tm}}$, $RN_{op_{tm}}$, $UN_{op_{tm}}$, $N_{C}$ and $C_{i}S_{4_{tm}}$). Then, CS extracts $RN_{sp_{l}}$, $UN_{sp_{l}}$, $RN_{op_{l}}$ and $UN_{op_{l}}$ from receiving parameters (such as $RN_{sp_{tm}}$). $UN_{sp_{l}}$ and $UN_{op_{l}}$ is used to retrieve $UN_{sp_{m}}$ and $UN_{op_{m}}$ from datasets. CS extracts $C_{i}S_{4}$, $SN_{C}$, $TS_{C_{i}}$ and checks timestamp. Then, CS computes Sigs ($CSS_{1}$, $CSS_{2}$ and $CSS_{3}$), and uses $CSS_{1}$ to extract original $C_{i}S_{1}$ and $C_{i}S_{2}$. After that, CS checks $CSS_{2}$ = $C_{i}S_{1}$ and $CSS_{3}$ = $C_{i}S_{2}$. If the Sigs are not identical, CS cancels the connection; otherwise, it moves to the next protocol.
    2. Second protocol as shown in Figure 10:
      * CS generates random secret ($SN_{CS}$) and new timestamp ($TS_{CS}$) between CS and AS. Then, CS computes the secret signature ($CSS_{4}$) to protect $SN_{CS}$. In addition, CS hides $C_{i}$’s parameters such as $N_{C}$ and $TS_{C_{i}}$ to use them with validation operations in AS and DS. In addition, all Sigs (such as $CSS_{2_{tm}}$) and PP (such as $N_{CS}$ and $TS_{CS_{tm}}$) are anonymously hidden by CS. At this point, CS sends XACML’s request to AS.
      * AS receives the request, cuts Sigs and PP. After that, AS extracts original parameters (such as $C_{i}S_{4}$ and $TS_{CS}$) and checks timestamp. AS computes $ASS_{1}$ (to extract $CSS_{2}$ and $CSS_{3}$) and computes $ASS_{2}$ and $ASS_{3}$ (to check $ASS_{2}$ = $CSS_{2}$ and $ASS_{3}$ = $CSS_{3}$). AS retrieves $RN_{op_{h}}$ and $UN_{op_{h}}$ from dataset (depending on $RN_{op_{m}}$ and $UN_{op_{m}}$) and computes $ASS_{4}$ to ensure $C_{i}$ request is legitimate after checks $ASS_{4}=C_{i}S_{4}$. AS uses the parts of external pseudonyms to specify $UR_{sp}$, $UR_{op}$, $CN_{sp}$ and $CN_{sp}$. AS retrieves Sigs of SP and OP ($S_{sp}$ and $S_{op}$) depending on the internal SP and OP. AS uses PDP1 engine to evaluate XACML’s request after adding $S_{sp}$ and $S_{op}$ to that request. AS specifies user’s policy in PAP and checks user’s attributes in PIP. PDP1 applies policy to get a decision (permit, deny, not applicable and indeterminate). If decision = “permit”, AS uses $UR_{sp}$ to specify user’s role (direct/indirect). If $UR_{sp}$ = direct, AS sends the data retrieval request by PRP to DS; if $UR_{sp}$=indirect, AS sends the Shamir request that contain at least 2 $SS_{s}$ to ensure legitimate indirect users. Otherwise AS sends reject response to $C_{i}$ by CS.
    3. Third protocol as shown in Figure 11:
      * Similarly, AS generates random secret ($SN_{AS}$) and timestamp ($TS_{AS}$) between AS and DS. AS computes $ASS_{5}$ to protect secret ($SN_{AS}$) between AS and DS. Additionally, AS computes $ASS_{6}$ to ensure legitimate PP ($RN_{op_{m}}$ and $UN_{op_{m}}$) in DS. All Sigs (such as $ASS_{6_{tm}}$) and PP (such as $TS_{AS_{tm}}$ and $SN_{AS_{tm}}$) are anonymously hidden by AS. Then, AS sends XACML’s request to DS.
      * DS receives the request, cuts Sigs and PP. After that, DS extracts original parameters (such as $C_{i}S_{4}$ and $SN_{AS}$) and checks timestamp. DS computes $DSS_{1}$ (to extract $ASS_{6}$) and retrieves $RN_{op_{h}}$ and $RN_{op_{m}}$ depending on $RN_{op_{l}}$. Then, DS computes $DSS_{2}$ and $DSS_{3}$ to check $DSS_{2}=ASS_{6}$ and $DSS_{3}=C_{i}S_{4}$. If AS’s parameters validated in DS correctly, DS computes timestamp ($TS_{DS}$) and signs patient’s data ($DSS_{4}$). All Sigs (such as $DSS_{4_{tm}}$) and PP (such as $TS_{DS_{tm}}$) are anonymously hidden by DS. At this point, DS sends the response to AS.
      * AS receives the response, extracts PP (such as $TS_{DS}$) and checks timestamp. AS tests the Sigs checking (such as $ASS_{6}=DSS_{2}$). Then, AS computes data signature ($ASS_{7}$) to check data integrity by $ASS_{7}=DSS_{4}$.
    4. Fourth protocol as shown in Figure 12:
      * AS prepares the response to CS by generating a new timestamp ($TS_{AS}$), hides data signature ($ASS_{7}$) with $ASS_{2}$, $ASS_{3}$, $C_{i}S_{4}$ and secret signature ($ASS_{1}$). AS hides PP and sends the response that contains decision and patient’s data to CS.
      * CS receives the response and extracts Sigs and PP. CS computes data signature ($DSS_{5}$) to check data integrity ($CSS_{5}=ASS_{7}$). Then, CS checks other Sigs ($CSS_{2}$, $CSS_{3}$ and $CSS_{4}$) with received Sigs ($ASS_{2}$, $ASS_{3}$ and $C_{i}S_{4}$) to ensure legitimacy of AS. CS prepares the response to $C_{i}$ by generating a new timestamp and hides data signature ($CSS_{5}$) with $CSS_{2}$, $CSS_{3}$, $C_{i}S_{4}$ and secret signature ($CSS_{1}$). CS sends the response to $C_{i}$.
      * $C_{i}$ receives the response, extracts PP and checks timestamp. $C_{i}$ computes data signature ($C_{i}S_{5}$) to check data integrity by $C_{i}S_{5}=CSS_{5}$. Then, $C_{i}$ extracts signatures ($CSS_{2}$, $CSS_{3}$, $CSS_{1}$ and $C_{i}S_{4}$) and checks them with original signatures ($C_{i}S_{1}$, $C_{i}S_{2}$, $C_{i}S_{3}$ and $C_{i}S_{4}$) respectively. $C_{i}$ uses $CSS_{2}$, $CSS_{3}$ and $CSS_{1}$ (secret signature between $C_{i}$ and CS) to check legitimacy of CS while uses $C_{i}S_{4}$ to check legitimacy of AS and DS. If all Sigs are validated, namely, authorised $C_{i}$ received securely correct data.
- **Authorisation protocols for indirect subjects and objects**
  Indirect user authorisation is an important process to secure sensitive patients’ data in the EHR stored in DS. PAX offers additional procedures to prevent the abuse of indirect user privileges.
  - **Prerequisite procedures**
    There are a set of steps that must be performed before authorisations are applied.
    1. Steps from 1 to 3 are similar to those for direct users.
    2. The Shamir scheme is used to generate the $SS_{s}$ from MS for the number of users, each $C_{i}$ has unique SS same length as MS, and authorised with two policies for each indirect user on AS. The policy evaluation process is also done with two, PDP1 and PDP2, evaluation engines. The use of two evaluation engines is very important in separating direct and indirect users and increasing security in the privacy of medical records.
    3. The PAX authorisation system identifies certain medical records (the patients’ history at a given time such as a year or more ago) for indirect users who can access them, as shown in Figure 13 (researcher case).
  - **Authorisation protocols**
    The following protocols detail how the indirect user obtains medical records in PAX. Figure 14 illustrates generally the authorisation of indirect users, while Figure 9, Figure 10, Figure 11 and Figure 12 and Figure 15 show the authorisation protocols of indirect users in PAX.
    1. The steps of the first and second protocols are similar to the ones of the direct users authorisation.
    2. Third protocol as shown in Figure 15:
      * AS computed MS previously by signing all users’ signatures. Then, AS computes Shamir scheme to generate $SS_{s}$ with the same number of users (each $C_{i}$ has one unique SS). In PAX, $C_{i}$ needs at least 3 $SS_{s}$ to generate original MS. In this protocol, AS generates a new timestamp and retrieves at least 2 $SS_{s}$. After that, AS hides $SS_{s}$ with $ASS_{2}$, $C_{i}S_{4}$, $S_{sp}$ and secret signature ($ASS_{1}$) as well as parameters (such as $TS_{AS_{tm}}$ and $UN_{sp_{tm}}$) are anonymously hidden. At this point, AS sends request to CS.
      * CS receives the request, extracts PP and checks timestamp. Then, CS removes the secret signature ($CSS_{4}$) and adds the secret signature ($CSS_{1}$) in $CSS_{2_{tm}}$. CS generates a new timestamp ($TS_{CS}$), hides PP and sends the request to $C_{i}$.
      * $C_{i}$ receives Shamir’s request, extracts PP and checks timestamp. Then, $C_{i}$ computes $C_{i}S_{6}$ to extract $SS_{s}$ and retrieves his SS. At the moment, $C_{i}$ can generate MS from Shamir ($C_{i}$’s SS||$SS_{s}$), hides MS with $C_{i}S_{6}$ and $C_{i}S_{3}$, generates timestamp and hides PP. At this point, $C_{i}$ sends the response to CS.
      * CS receives the response, extracts PP and checks timestamp. Also, CS removes $CSS_{1}$ and adds $CSS_{4}$ in $C_{i}S_{6_{tm}}$. CS generates a new timestamp, hides PP and sends the response to AS.
      * AS receives Shamir response, extracts PP and checks timestamp. Then, AS extracts the received MS and checks it with the saved original MS. After that, AS retrieves $C_{i}$’s SS depending on $S_{sp}$($UR_{sp}\left|\right|CN_{sp}$) and assigns the request (SS,$S_{op}$) to PDP2 . AS specifies policy depending on policy’s ID (Shamir||SP), checks attributes in PIP and PDP2 applies policy in PAP to produce the decision. If the decision is “permit”, AS creates a data retrieval request by PRP to DS; otherwise AS sends reject response to $C_{i}$ by CS.
    3. The fourth and fifth protocols are similar to the third and fourth ones respectively in direct user authorisation. DS sends the response to the $C_{i}$ by AS and CS. If $C_{i}$ is an advisor, relative, or emergency doctor, $C_{i}$ will receive specific patient’s data; otherwise, if $C_{i}$ is researcher doctor, $C_{i}$ will receive a set of medical records.

## 5. Discussion

In this Section, we discuss users scenarios and security analysis in PAX and demonstrate PAX’s ability to protect patients’ data during security and privacy implementation. In addition, the use of formal tools in the PAX security analysis is to prove security measures in repelling healthcare risks.

### 5.1. Direct and Indirect Users Scenarios in PAX

**Figure 16:**
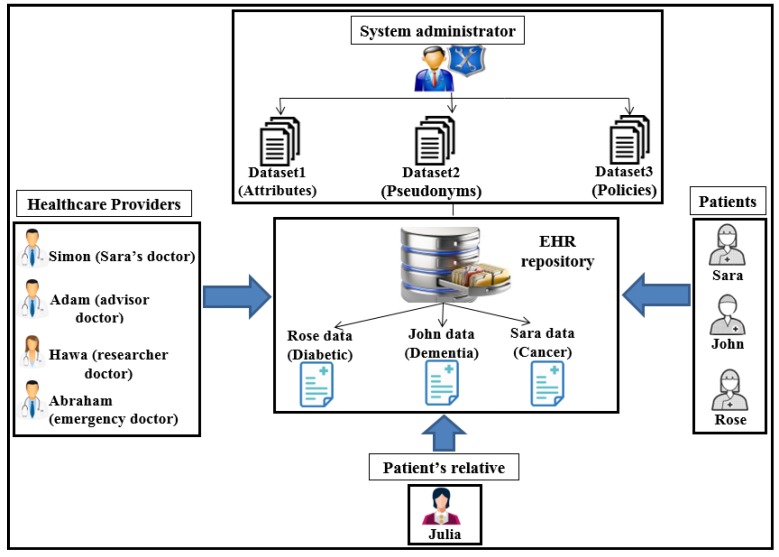
Users’ scenarios in PAX.

This Section illustrates four case scenarios in PAX that involve obtaining access to medical records in the EHR. We present our perspective of securing the privacy of patients’ data through the integration of anonymity, pseudonym, and XACML in our project. To provide user scenarios, we impose a number of EHR users with the PAX system, as shown in Figure 16. The patient may suffer from many diseases such as diabetes, dementia, cancer, addiction, blood pressure, and heart disease, which means that the patient is associated with more than one doctor. The patient does not want other healthcare providers to access his/her personal information because of embarrassment or his/her psychological state. In addition, the doctor has treated a set of patients. Therefore, ensuring privacy in non-disclosure of personal information to patients requires each indirect user to apply HIPAA standards.

**Figure 17:**
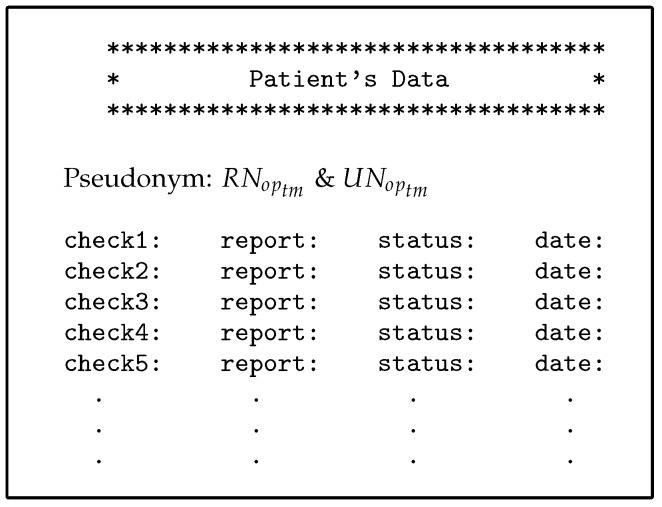
Part of Sarah’s data.

Assume we have three patients, Sara, John, and Rose, who suffer from diseases such as cancer, dementia, and diabetes respectively. Each disease requires a different level of care. For instance, a patient suffering from dementia needs a family member who assists with all of the patient’s tasks and is able to access all of the patient’s data. We assume that Julia is one of John’s relatives. In addition, there is a group of healthcare providers, including Simon, Adam, Hawa, and Abraham, who want access to patients’ medical records. These users can have different roles; for example, Adam may have the roles of advisor and doctor, and Abraham may be a doctor and an emergency doctor. Different user roles can be a major reason for breaching the privacy of medical records. Users such as patients (Sara and Rose) and the physician (Simon) need direct authorisation to EHR data because of persistent and regular requests to access the repository. For example, Simon is the general practitioner (GP) for Sara and needs to access her data every day or even more than once a day (under the PAX system, Sara’s data is private in data access requests by both Sara and Simon, as shown in Figure 17).

1. The first scenario (advisor): Simon needs a consultant (such as Adam) to diagnose Sara’s disease or to submit treatment suggestions (after taking Sara’s consent to seek specialist advice). Adam is not associated with Sara permanently and continuously and does not need Sara’s personal information; he only needs certain details of the patient’s data and medical reports. Therefore, in PAX, Adam needs to enter his name (Adam), the name of the doctor (Simon), and Sara’s pseudonym to access Sara’s data; he does not need to know Sara’s real attributes. Figure 17 shows Sara’s data, which can be obtained by Simon and Adam. We note from Figure 17 that the data received does not contain any of Sara’s attributes, and Adam does not use any real attributes for Sara, which means that PAX provides a high level of security and privacy that can prevent external and internal attacks.
2. The second scenario (relative of a patient): Because the patient (John) suffers from dementia, he is unable to perform his duties. John needs a family helper (such as Julia) to access his medical data without misuse or to bypass these privileges to other medical records. Julia needs a request that contains her Sigs and John’s pseudonym to be considered a legitimate user in the system but is not authorised to access John’s data until the CS and AS complete the third authorisation protocol with the Shamir scheme.
3. The third scenario (researcher): Hawa is a researcher and tries to access the server’s repository to use EHR in evaluating a medical study to develop a disease treatment. The researcher needs access to medical records sporadically and not permanently. The researcher is not associated with a particular patient and needs access to a set of the patients’ data. In addition, this indirect user does not need access to the patients’ attributes. Figure 13 shows a set of medical records obtained by Hawa in the case of authorisation without using any of the patients’ real attributes.
4. The fourth scenario (emergency doctor): When Rose’s health has deteriorated significantly and suddenly, her doctor is not available for some reason. Rose needs an emergency doctor to treat and assess her condition quickly (e.g., Abraham). The emergency doctor needs to access Rose’s data without accessing personal information. In an emergency, access to a patient’s data does not require the patient’s consent. Abraham should not know any secrets healthcare providers have used to authorise access to Rose’s data.

PAX provides security and privacy for all previous scenarios; indirect users cannot access the patient’s personal information because it is separate and completely hidden from the data. As a result, the user can retrieve this data to improve healthcare without penetrating the repository in DS.

### 5.2. Security Analysis

Security and privacy mechanisms in PAX have been evaluated under theoretical analysis, BAN logic and AVISPA tool.

#### 5.2.1. Theoretical Security Analysis

Organizational and managerial features are important in healthcare systems, but the key player in applying these systems is the use of security and privacy mechanisms for patient records [11]. Medical records in the EHR are sensitive data and require security mechanisms to protect their privacy from attackers. In addition, the different levels and privileges of healthcare providers make the development of security mechanisms and authorisation models very difficult [4]. Moreover, applying privacy to medical records (EHR) requires the use of access models in the authorisation of users. Integrating RBAC and ABAC gives more powerful features to PAX users. The result is an access control model based on roles and attributes that handle users’ requests at the coarse-grained and fine-grained levels. To increase security and privacy in the authorisation model, we have added a set of mechanisms to hide and separate personal information about data. The PAX system ensures that legitimate users access their specific data and, on the other hand, the privacy of medical records is maintained. Any healthcare system should support the basic security features of confidentiality, integrity, and availability (C.I.A.) [7], and there is a set of security features included in PAX.

1. Integrity and non-repudiation of requests
  User requests and policies need protection from change or repudiation. We used the ECDSA algorithm to sign user attributes. Any change in the Sigs will be detected in the server because the server checks the users’ requests before authorising access to the data. In addition, the signatory party cannot deny its Sig. These features make the system immune against changing attacks such as MITM.
2. Authentication and authorisation of requests
  Each EHR requires authentication and authorisation properties to protect medical records from unauthorised access. We applied ECDSA to the XACML v3.0 to support these properties in PAX. The use of Sigs in XACML between the $C_{i}$ and the CS, AS and DS support user authentication in addition to the use of policies and rules to identify authorised users and the level of access granted to them by providing anti privileged insider and authorisation policies.
3. Confidentiality and anonymization
  One of the security features of hiding information is confidentiality. We applied ECDSA to add confidential requests to subjects and objects, and we added a Shamir scheme (backup or fail-open mechanism) to provide anonymity of $SS_{s}$ to users of the EHR system. This process prevents the attacker from seeing explicit attributes and does not allow the hacker to know the user-configured SS for any healthcare provider. A Shamir scheme ensures the anonymity of the Sig. This backup mechanism enables indirect users to access protected health information (PHI) with privacy and security.
4. Pseudonymization
  A patient’s privacy requires the separation of personal information from the patient’s data. Pseudonym prevents the intruder from knowing the data of any of the patients. PAX supports pseudonym in both subjects’ and objects’ attributes using pseudonyms for real attributes. This feature supports the privacy of a patient’s data.
5. Audit and activities
  PAX records all user activities (requests and responses) to access medical records. It monitors user activities, including the number of access times, the result of the decision, and the amount of data required. The audit process is important for any healthcare system in determining users’ activities. PAX stores and organizes requests and responses for each user (patient, doctor, advisor, relative, researcher, and emergency doctor) separately to facilitate the management of these activities.

There is a range of attacks that pose a serious risk to any healthcare system. PAX’s security mechanisms act as countermeasures (as shown in Table 3) against known attacks.

1. Availability attacks
  The server is vulnerable to the denial of service (DoS) attacks that are intended to disable the service. In PAX, the indirect user creates a random Sig based on $SS_{s}$ provided by healthcare providers. The attacker cannot use the same $SS_{s}$ because the CS and AS will ignore the request. The abundance of medical records is critical to healthcare providers’ flexible access. Therefore, supporting robustness in any healthcare system depends on preventing DoS attacks. Although the PAX system limits the risk of DoS attacks and provides successfully anti DoS, it does not do so fully because the attacker can still send requests without penetrating the patient’s personal information and data.
2. Data and policies datasets attacks
  The data in the single server is considered an attractive treasure for attackers. In addition, policies contain the attributes and roles of users, which can assist attackers in carrying out an attack to recognise and access patients’ data. In PAX, even if the attacker obtained a patient’s data, the data would not be useful because both the stored and movable data would have a pseudonym. In addition, the data is stored (on DS) separately from policies (on AS) as well as PAX policies are associated with pseudonym and anonymity (both CS and DS do not have real attributes datasets for users), which prevent attackers from revealing subjects’ and objects’ attributes. Consequently, PAX provides effectively authorisation policies and anti datasets attacks.
3. Modification attacks on requests
  Users’ requests from clients to server in PAX are fully protected from modification. PAX uses random nonces and Sigs to detect changing operation by intruders. Thus, PAX fully is secure against MITM attacks.
4. Replay attacks
  The intruder cannot resend authorisation request to the network later because PAX produces a new timestamp ($TS_{C}$, $TS_{CS}$, $TS_{AS}$, and $TS_{DS}$) between PAX’s entities. Therefore, PAX withstands securely against replay attacks.
5. Unauthorised access attacks
  User access to a repository depends on authorisation policies. We use XACML v3.0 to create user policies. Integrating RBAC and ABAC into XACML prevents internal/external unauthorised users from accessing patients’ data. Thus, PAX reliably provides anti privileged insider depending on users’ role and attributes.
6. Traffic analysis attacks
  To perform this attack, the hacker must analyse either the requests or the data moving between the source and the target. In PAX, if we assume that the attacker has some attributes (such as the name) and expects a specific patient, the attacker cannot use a keyword (name) and analyse it with multiple requests or medical records, even if it is the same user, to reveal its real attributes; the attacker cannot identify this data for a particular patient. Using pseudonym and anonymity prevents attackers from tracking/leaking traffic. For example, if advisor1 and advisor2 want patient1 data, the generated requests will be different. This prevents the parsing of requests. As a result, PAX supports anonymity, pseudonym, anti traceability and anti leakage features.
7. Impersonation attacks
  The intruder cannot impersonate PAX’s entities ($C_{i}$, CS, AS and DS) because PAX uses secret nonces ($SN_{C}$, $SN_{CS}$ and $SN_{AS}$) and secret Sigs among entities to support mutual authentication and prevent impersonation attacks. Thereupon, PAX resists impersonation attacks of the fake client/server.
8. Timing attacks
  This attack exploits the security procedure while calculating the time period for security operations (such as encryption and signing). PAX prevents these attacks because when the attacker gets multiple requests for the same user, the attacker will find that these requests contain different Sigs, and the attacker does not have the parameters to generate the Sig. In addition, ECDSA’s Sigs with 256-bit is resistant to timing attacks. Hence, PAX robustly prevents timing attacks.

#### 5.2.2. Proof of PAX Security Protocol

To verify request source, freshness and legitimacy of entity in PAX, we have used Burrows, Abadi and Needham (BAN) logic that depends set of rules such as seeing (SR), message meaning (MMR), nonce verification (NVR), jurisdiction (JR), freshness conjuncatenation (FCR) and shared secret (SSR) (details about BAN’s notations and rules is available in [43,44,45]). With BAN, we prove that each entity in PAX deals with other legitimate entity when transferring the message (M) from the indirect user to the servers and vice versa. BAN structures consist of goals (G), idealized form, hypotheses (H) and proofs of goals by applying rules and hypotheses.

- **Goals**: PAX must provide the following goals to securely exchange messages among PAX entities.
  - **G1**:$C_{i}|\equiv C_{i}\overset{C_{i}S_{3}}{⇌}CS$
  - **G2**:$CS|\equiv C_{i}\overset{CSS_{1}}{⇌}CS$
  - **G3**:$CS|\equiv CS\overset{CSS_{4}}{⇌}AS$
  - **G4**:$AS|\equiv CS\overset{ASS_{1}}{⇌}AS$
  - **G5**:$AS|\equiv DS\overset{ASS_{5}}{⇌}AS$
  - **G6**:$DS|\equiv AS\overset{DSS_{1}}{⇌}DS$
- **Idealized form**: The messages (M) are represented in a BAN formula.
  - **M1**: $C_{i}\to CS$: $C_{i}S_{1_{tm}}||RN_{sp_{tm}}||UN_{sp_{tm}}\left|\right|N_{C}||TS_{C_{tm}}\left|\right|SN_{C_{tm}}$, $C_{i}S_{2_{tm}}||RN_{op_{tm}}||UN_{op_{tm}}\left|\right|N_{C}\left|\right|C_{i}S_{4_{tm}}$: ${\langle SN_{C}\rangle }_{C_{i}S_{3}}$
  - **M2**: CS→AS: $CSS_{2_{tm}}||RN_{sp_{tm}}||UN_{sp_{tm}}\left|\right|N_{CS}||TS_{C_{tm}}\left|\right|$$TS_{CS_{tm}}\left|\right|SN_{CS_{tm}}$, $CSS_{3_{tm}}\left|\right|RN_{op_{tm}}$$||UN_{op_{tm}}$$\left|\right|C_{i}S_{4_{tm}}$: ${\langle SN_{CS}\rangle }_{CSS_{4}}$
  - **M3**: AS→CS: $ASS_{2_{tm}}||UN_{sp_{tm}}\left|\right|TS_{AS_{tm}}$: ${\langle SN_{CS}\rangle }_{ASS_{1}}$
  - **M4**: $CS\to C_{i}$: $CSS_{2_{tm}}||UN_{sp_{tm}}\left|\right|TS_{CS_{tm}}$: ${\langle SN_{C}\rangle }_{CSS_{1}}$
  - **M5**: $C_{i}\to CS$: $C_{i}S_{6_{tm}}||UN_{sp_{tm}}\left|\right|TS_{C_{tm}}$: ${\langle SN_{C}\rangle }_{C_{i}S_{3}}$
  - **M6**: CS→AS: $CiS_{6_{tm}}||UN_{sp_{tm}}\left|\right|TS_{CS_{tm}}$: ${\langle SN_{CS}\rangle }_{CSS_{4}}$
  - **M7**: AS→DS: $ASS_{6_{tm}}||RN_{op_{tm}}||UN_{op_{tm}}||SN_{AS_{tm}}\left|\right|TS_{C_{tm}}$$||TS_{AS_{tm}}\left|\right|C_{i}S_{4_{tm}}$: ${\langle SN_{AS}\rangle }_{ASS_{5}}$
  - **M8**: DS→AS: $DSS_{2_{tm}}||DSS_{4_{tm}}||UN_{op_{tm}}||TS_{DS_{tm}}\left|\right|C_{i}S_{4_{tm}}$||"Data": ${\langle SN_{AS}\rangle }_{DSS_{1}}$
  - **M9**: AS→CS: $ASS_{2_{tm}}||ASS_{3_{tm}}||UN_{sp_{tm}}||TS_{AS_{tm}}\left|\right|$"Decision & Data": ${\langle SN_{CS}\rangle }_{ASS_{1}}$
  - **M10**: $CS\to C_{i}$: $CSS_{2_{tm}}||CSS_{3_{tm}}||UN_{sp_{tm}}||TS_{CS_{tm}}\left|\right|$"Decision & Data": ${\langle SN_{C}\rangle }_{CSS_{1}}$
- **Hypotheses**: Sets of hypotheses to analyse PAX’s security.
  - **H1**: $C_{i}|\equiv \#\left(SN_{C}\right)$.
  - **H2**: $C_{i}|\equiv \#\left(TS_{C_{i}}\right)$.
  - **H3**: $CS|\equiv \#(SN_{C})$.
  - **H4**: $CS|\equiv \#(TS_{CS})$.
  - **H5**: $CS|\equiv \#(SN_{CS})$.
  - **H6**: $AS|\equiv \#(SN_{CS})$.
  - **H7**: $AS|\equiv \#(TS_{AS})$
  - **H8**: $AS|\equiv \#(SN_{AS})$.
  - **H9**: $DS|\equiv \#(SN_{AS})$.
  - **H10**: $DS|\equiv \#(TS_{DS})$.
  - **H11**: $CS|\equiv C_{i}⟹SN_{C}$.
  - **H12**: $AS|\equiv CS⟹SN_{CS}$.
  - **H13**: $DS|\equiv AS⟹SN_{AS}$.
  - **H14**: $C_{i}|\equiv C_{i}\overset{KCS_{pu}}{\mapsto }CS$.
  - **H15**: $CS|\equiv CS\overset{KC_{pu}}{\mapsto }C_{i}$.
  - **H16**: $CS|\equiv CS\overset{KAS_{pu}}{\mapsto }AS$.
  - **H17**: $AS|\equiv AS\overset{KCS_{pu}}{\mapsto }CS$.
  - **H18**: $AS|\equiv AS\overset{KDS_{pu}}{\mapsto }DS$.
  - **H19**: $DS|\equiv DS\overset{KAS_{pu}}{\mapsto }AS$.
- **Proofs**: We have used BAN logic to prove goals based on rules and hypotheses.
  - **M1**: $C_{i}\to CS$:
    * **SR**:
      S1: CS⊲ M1
    * **MMR**:
      Using MMR, S1 and H14, S2: $CS|\equiv C_{i}|∼SN_{C}$
    * **NVR and FCR**:
      Using NVR, FCR, S2, H1 and H2, S3: $CS|\equiv C_{i}|\equiv SN_{C}$
    * **JR**:
      Using JR, S3 and H11, S4: $CS|\equiv SN_{C}$
    * **SSR**:
      Using SSR, S3, H1 and H2, S5: $CS|\equiv C_{i}\overset{CSS_{1}}{⇌}CS$ (**G2**)
  - **M2**: CS→AS:
    * **SR**:
      S6: AS⊲ M2
    * **MMR**:
      Using MMR, S6 and H16, S7: $AS|\equiv CS|∼SN_{CS}$
    * **NVR and FCR**:
      Using NVR, FCR, S7, H4 and H5, S8: $AS|\equiv CS|\equiv SN_{CS}$
    * **JR**:
      Using JR, S8 and H12, S9: $AS|\equiv SN_{CS}$
    * **SSR**:
      Using SSR, S8, H4 and H5, S10: $AS|\equiv CS\overset{ASS_{1}}{⇌}AS$ (**G4**)
  - **M3**: AS→CS:
    * **SR**:
      S11: CS⊲ M3
    * **MMR**:
      Using MMR, S11 and H17, S12: $CS|\equiv AS|∼SN_{CS}$
    * **NVR and FCR**:
      Using NVR, FCR, S12, H6 and H7, S13: $CS|\equiv AS|\equiv SN_{CS}$
    * **JR**:
      Using JR, S13 and H12, S14: $CS|\equiv SN_{CS}$
    * **SSR**:
      Using SSR, S13, H6 and H7, S15: $CS|\equiv CS\overset{CSS_{4}}{⇌}AS$ (**G3**)
  - **M4**: $CS\to C_{i}$:
    * **SR**:
      S16: $C_{i}⊲$ M4
    * **MMR**:
      Using MMR, S16 and H15, S17: $C_{i}|\equiv CS|∼SN_{C}$
    * **NVR and FCR**:
      Using NVR, FCR, S17, H3 and H4, S18: $C_{i}|\equiv CS|\equiv SN_{C}$
    * **JR**:
      Using JR, S18 and H11, S19: $C_{i}|\equiv SN_{C}$
    * **SSR**:
      Using SSR, S18, H3 and H4, S20: $C_{i}|\equiv C_{i}\overset{C_{i}S_{3}}{⇌}CS$ (**G1**)
  - **M5**: $C_{i}\to CS$: Similar to the M1 (**G2**)
  - **M6**: CS→AS: Similar to the M2 (**G4**)
  - **M7**:AS→DS:
    * **SR**:
      S21: DS⊲ M7
    * **MMR**:
      Using MMR, S21 and H18, S22: $DS|\equiv AS|∼SN_{AS}$
    * **NVR and FCR**:
      Using NVR, FCR, S22, H7 and H8, S23: $DS|\equiv AS|\equiv SN_{AS}$
    * **JR**:
      Using JR, S23 and H13, S24: $DS|\equiv SN_{AS}$
    * **SSR**:
      Using SSR, S23, H7 and H8, S25: $DS|\equiv DS\overset{DSS_{1}}{⇌}AS$ (**G6**)
  - **M8**: DS→AS:
    * **SR**:
      S26: AS⊲ M8
    * **MMR**:
      Using MMR, S26 and H19, S27: $AS|\equiv DS|∼SN_{AS}$
    * **NVR and FCR**:
      Using NVR, FCR, S27, H9 and H10, S28: $AS|\equiv DS|\equiv SN_{AS}$
    * **JR**:
      Using JR, S28 and H13, S29: $AS|\equiv SN_{AS}$
    * **SSR**:
      Using SSR, S28, H9 and H10, S30: $AS|\equiv AS\overset{ASS_{5}}{⇌}DS$ (**G5**)
  - **M9**: AS→CS: Similar to the M3 (**G3**)
  - **M10**: $CS\to C_{i}$: Similar to the M4 (**G1**)

#### 5.2.3. Proof of PAX Security Mechanism

In this Section, we simulate the PAX scheme using the AVISPA tool to test and analyse that user authorisation information is safe during its transition between PAX entities and immune against active and passive attacks.

**Figure 18:**
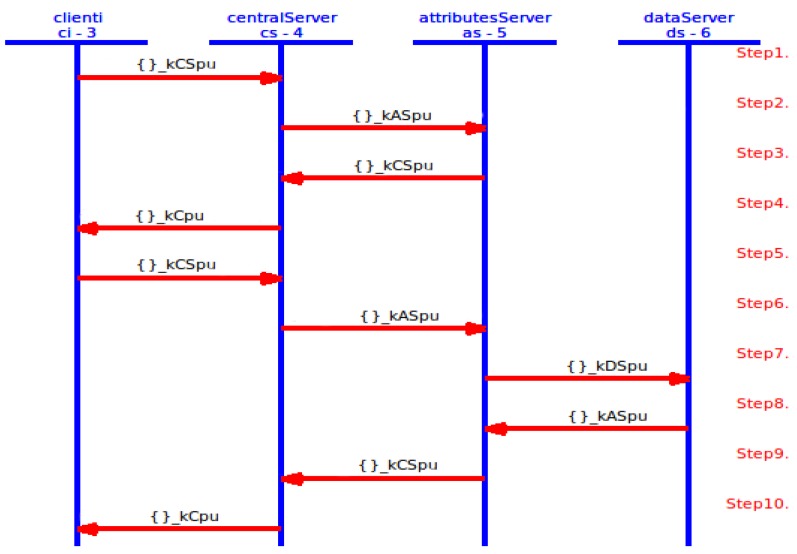
PAX’s framework in AVISPA.

**Figure 19:**
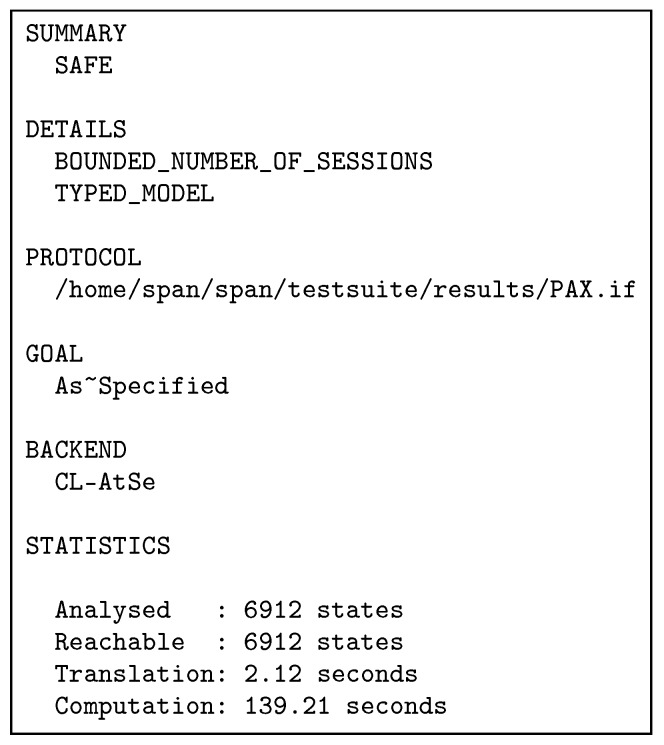
PAX’s result in CL-AtSe.

**Figure A1:**
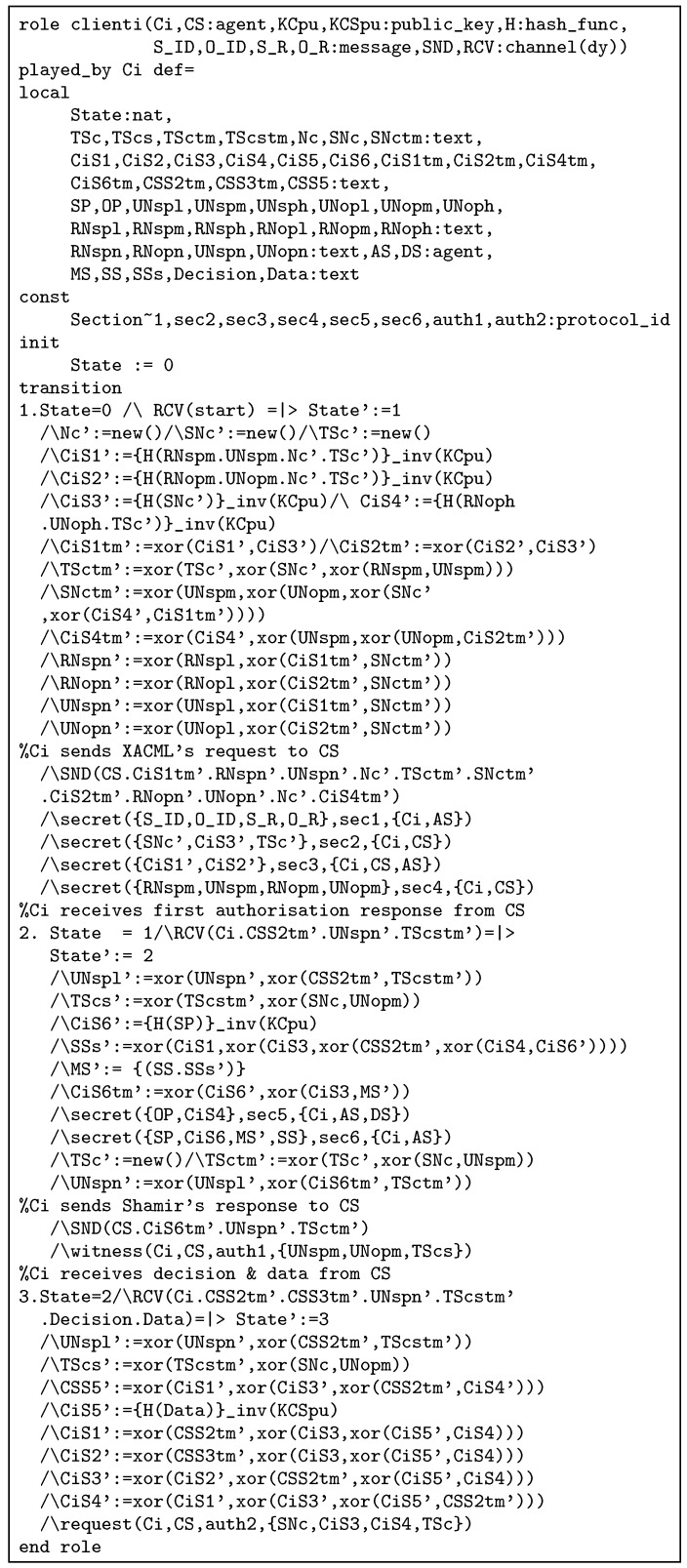
$C_{i}$ role of PAX in high-level protocol specification language (HLPSL).

**Figure A4:**
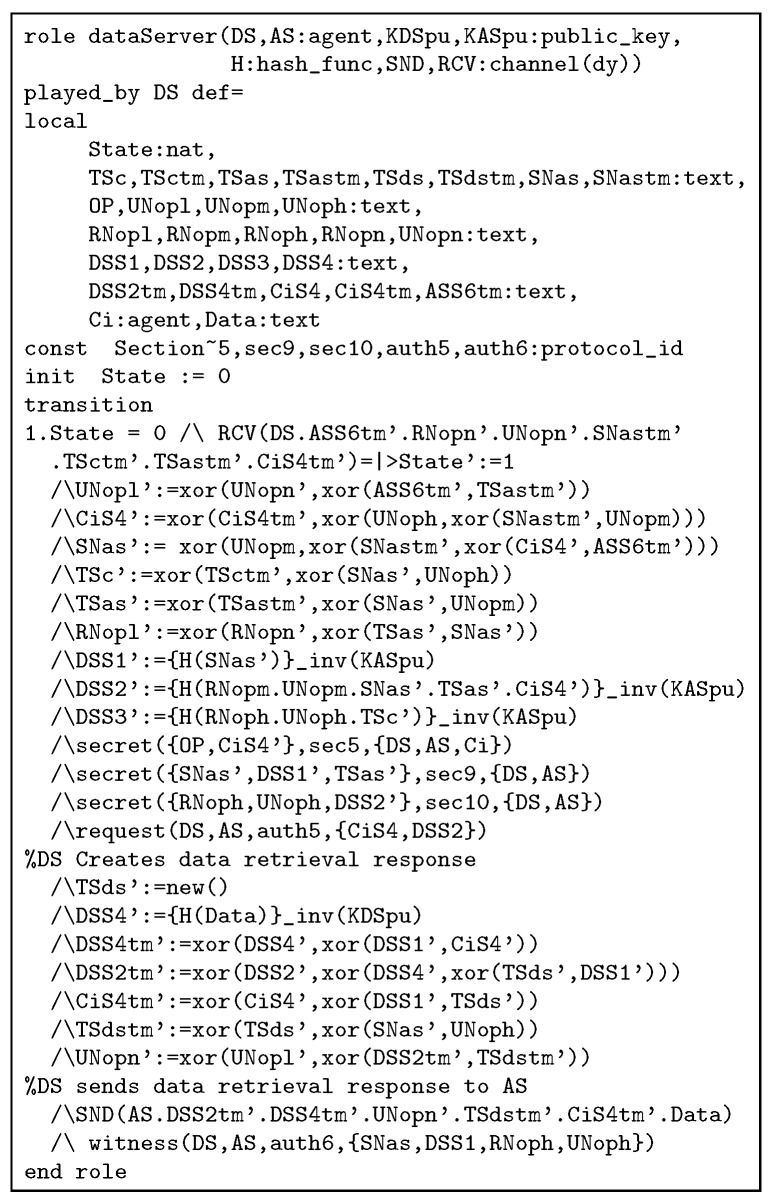
DS role of PAX in HLPSL.

**Figure A5:**
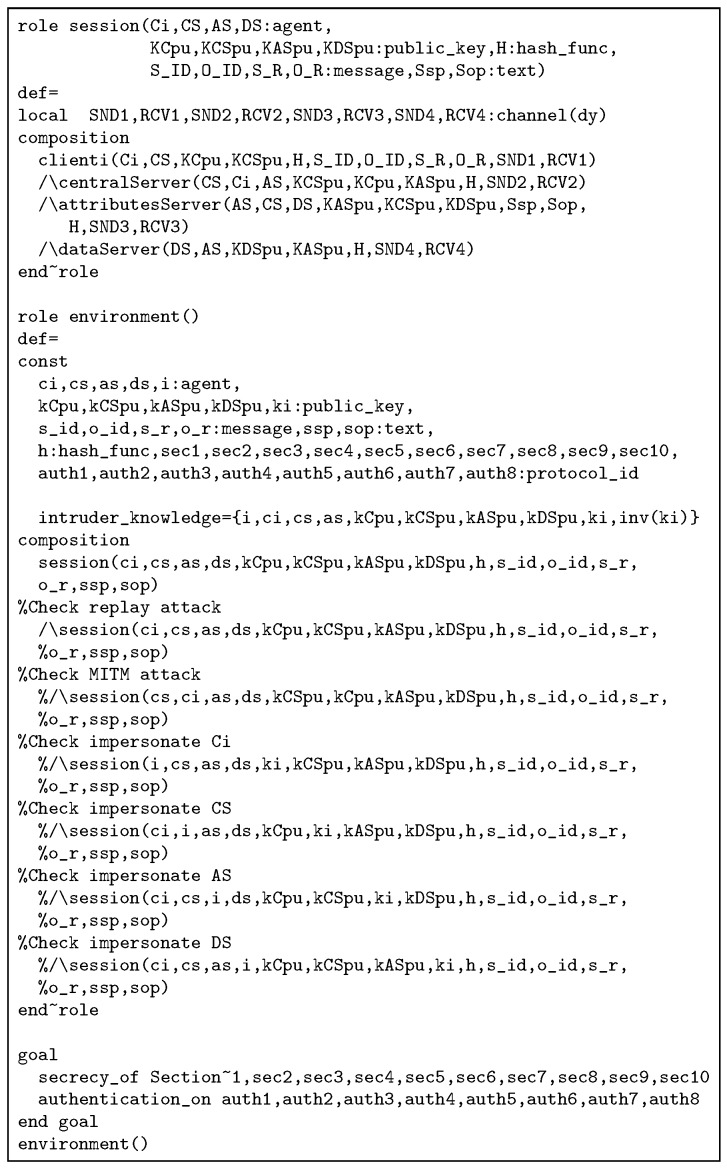
Session, environment, and goal role of PAX in HLPSL.

- AVISPA Briefly
  After designing any authorisation scheme, this scheme should be validated and its accuracy verified under a security analysis tool such as AVISPA to analyse, trace, observe and test the possibility of threat experimentally. The AVISPA tool is a push-button, testing/proofing model and is used directives and expressive terms intermediate format (IF) and output format (OF) to achieve simulation of security analysis [3,46,47]. AVISPA is a formal tool for analysing security schemes and applied by researchers to evaluate recent security protocols [48,49,50,51]. This tool is based on the Dolev-Yao (dy) model in analysis protocols during the transmission of information in the communication channels. It provides many features to analyse security schemes, such as a practical assessment of error detection and tracking, statistics, accurate results, testing of many techniques on the one protocol, ease of use, robustness of this tool to implement security protocols [46]. This tool deals with high-level protocol specification language (HLPSL) and 4 backends such as Constraint-Logic-based Attack Searcher (CL-AtSe) to extract the results of the scheme analysis (more detailed information about the HLPSL language and the description of the AVISPA tool is available in [46,52]).
- PAX with AVISPA
  In terms of HLSPL with AVISPA, PAX consists of four core (essential) roles: client ($C_{i}$), centralServer (CS), attributesServer (AS) and dataServer (DS). In addition, there are supporting roles such as session, and environment, goal specification section. Essential roles include a transition section (to specify the sequence of communication operations in network framework). Supporting roles include a composition section (to specify the linking of sessions and roles). PAX depends on asymmetric cryptography by using ECDSA with public keys ($KC_{pu}$, $KCS_{pu}$, $KAS_{pu}$ and $KDS_{pu}$) to perform security requirements (integrity, authentication and non-repudiation). Moreover, PAX applies nonces ($SN_{C}$, $SN_{CS}$, $SN_{AS}$ and $SN_{DS}$) to support anonymity and timestamps ($TS_{C}$, $TS_{CS}$, $TS_{AS}$ and $TS_{DS}$) to support freshness. Authorisation process for indirect users is illustrated by the HLPSL scripts in Figure A1–Figure A4 (in Appendix A). Each role consists of the number of transitions, the receiving process (RCV), the sending process (SND), the sender’s claim process of fresh value and correct (witness), the validation process in receiver for the sender’s claim (request), the process of creating a fresh value for the nonce and timestamp (new) and the use of the private key (_inv) in PAX’s entities. At first, $C_{i}$ receives the start signal as in Figure A1, then the SND and RCV operations continue until the authorisation process is completed as in Figure 18.
  Figure A5 shows the roles of session, environment, and goal section. In the session role, a composition process has been performed for the four roles (clienti, centralServer, attributeServer and dataServer) and specifies the send and receive channels in the Dolev-Yao model. In the environment role, the PP, the goals specified in the goal section, and the known information for the intruder (intruder_knowledge) have been defined. In this role, one or more sessions are composed, and we tested our scheme with sessions for replay, MITM, and impersonating entity attacks. We assumed that an intruder (*I*) creates a public key (ki) and has knowledge of the public keys (kCpu, kCSpu, and kASpu) of PAX’s entities in the network. Intruder attempts to resend legitimate user requests later, intercepts/modifies these requests, or impersonates the connecting entities using *i* constant rather than ci, cs, as and ds. The results displays that these attacks cannot penetrate the security goals in our scheme. Goal section describes verified goals in PAX, and provides 10 goals of secrecy (such as S_ID, O_ID, S_R and O_R represent the first secret (sec1) and known only for both ci and cs) and eight goals of authentication (such as UNspm, UNopm and TScs represent the first authentication between ci and cs).
- Simulation Result
  In this section, the simulation result is based on CL-AtSe backend in the AVISPA. Figure 19 displays the simulation result with the CL-AtSe backend, PAX clearly and accurately achieves the SAFE result in the SUMMARY section, bounded number of sessions in the DETAILS section, the goals of the scheme achieved (as_specified) in the GOAL section as well as statistical numbers such as time, number of nodes, and analysed states in the STATISTICS section. Based on this result, we note that our scheme is capable of preventing passive and active attacks such as replay, MITM, and impersonating, and that the goals of the scheme in Figure A5 successfully prevented the violation of legitimate users information in the network authorisation.

## 6. Comparison of Our Study with Other Research

In this section, we explain how our project addresses the gaps in related works [2,6,8,9,16,17,18]. PAX has not suffered from PERMIS’s problems [16] because each request to the healthcare provider has been signed randomly with the ECDSA algorithm, which includes both the roles ($RN_{s}$) and the pseudonyms ($UN_{s}$). In PAX, the policies are stored on the attributes server as Sigs and pseudonym rather than as explicit attributes in XACML (each user in PAX has attributes separate from other users that prevent the inheritance of attributes). Compared with [8], PAX has solved all requests standardization and structure problems by including XACML v3.0 and ECDSA. XACML v3.0 offers standardization, and generic and rich policy language and is unified with the context of subjects’ requests. It does not have problems converting requests from different sources. We also use ECDSA to generate very small keys relative to RSA to improve server performance. Furthermore, PAX does not need the keys complexity in PIPE [6] because XACML has the flexibility to handle practitioners and patients’ requests and we use only one high-performance signature algorithm. In our project, all the attributes in the requests and policies are not clearly written as in [2]. Moreover, data is anonymous to the patient when the data is transferred from the source to the target because it is linked with a random pseudonym.

Instead of one situation (emergency) as in HCPP, our project used several realistic situations such as doctor advisors, physician researchers, emergency doctors, and patients’ relatives for healthcare users and used the XACML v3.0 policy language, which is effective for authorising users. Our project does not require continuous mining [9] of patient data but relies on an internal pseudonym to access medical records. XACS [17] offers protection only against external attacks, whereas PAX offers protection against internal and external attacks by preventing attackers from identifying the personal information of legitimate users or patients’ data. Finally, The access control model in [18] deals with real attributes, whereas PAX integrates signatures and pseudonyms within XACML’s policies and requests to prevent the exchange of users’ attributes clearly during the authorisation process in healthcare applications [18]. Table 3 compares the security features provided in PAX and related works.

## 7. Conclusions and Future Work

The security and privacy of medical records have become essential requirements for the establishment of any healthcare system in recent years. To ensure the provision of security and privacy, this paper proposes a PAX authorisation system that supports pseudonym, anonymity and XACML. Specifically, the proposed system uses a random pseudonym to separate personal information about patients’ data, anonymity to hide subjects’ information, and XACML to create distributed access control policies to authorise subjects’ requests to objects’ records in EHR. Different from a large amount of theoretical investigation in the existing literature, this paper achieves the security and privacy preservation by utilizing the pseudonym and anonymity techniques, which can reduce the unnecessary time consumption and the burden on the server. Security analyses using the theoretical method or formal methods (BAN and AVISPA) demonstrate that PAX is safe, maintains the privacy of healthcare users and alleviates the risk of penetrating compared to existing research. We believe that the PAX system provides a security high-level that maintains patients’ privacy, and the system especially protects patients’ information from indirect users (advisors, patients’ relatives, researchers, and emergency doctors), who have been considered a serious security threat to any healthcare system because they can carry out internal attacks using the privileges granted to them. To further develop the proposed PAX system, we intend to add some features to support security and privacy in EHR.

1. PAX requires an authentication mechanism that is more stringent to identify legitimate users in the network and prevent counterfeit requests. We intend to use a one-time password based on users’ attributes, temporary parameters, and Sigs to support the authentication of legitimate users in PAX.
2. Patients’ data requires devices (such as WSN) to be aggregated accurately and continuously and sent to the CS and DS. However, data collection and storage on the server requires security mechanisms.
3. We will focus on patients’ data without the use of cryptographic mechanisms in examining the patients’ daily condition, use patients’ real data to test PAX with large data, and allow PAX to distinguish between patients’ history, daily status, and purpose of data access. We will also encrypt the patients’ old medical records (within a certain period) that are not frequently retrieved by healthcare providers in a manner that does not affect the efficiency of the server in providing the service to users.
4. We will investigate the application of a light hash algorithm to generate patients’ pseudonyms, which support increased randomization while maintaining system performance as an additional security measure to protect the privacy of medical records in EHR.
5. We intend to build an evaluation system to assess PAX in the exchange of requests among network entities $C_{i}$, CS, AS and DS in terms of authorisation request delay, cost of signature and verification, storage and throughput.

## Figures and Tables

**Table 1:** Internal and external pseudonyms of users.

Users	UR	CN	Internal Pseudonym	RN	UN	External Pseudonym
patient	*p*		$p_{1}\;...\;p_{n}$			
doctor	*d*		$d_{1}\;...\;d_{n}$			
advisor	*a*		$a_{1}\;...\;a_{n}$			
relative	pr	1...n	$pr_{1}\;...\;pr_{n}$	1...n	1...n	1...n
researcher	*r*		$r_{1}\;...\;r_{n}$			(48-bit)
emergency	*e*		$e_{1}\;...\;e_{n}$			
Shamir	-		-			

**Table 2:** Parts of SP and OP.

SP						OP					
${\mathrm{RN}}_{\mathrm{sp}}$			${\mathrm{UN}}_{\mathrm{sp}}$			${\mathrm{RN}}_{\mathrm{op}}$			${\mathrm{UN}}_{\mathrm{op}}$		
$RN_{sp_{l}}$	$RN_{sp_{m}}$	$RN_{sp_{h}}$	$UN_{sp_{l}}$	$UN_{sp_{m}}$	$UN_{sp_{h}}$	$RN_{op_{l}}$	$RN_{op_{m}}$	$RN_{op_{h}}$	$UN_{op_{l}}$	$UN_{op_{m}}$	$UN_{op_{h}}$

**Table 3:** Comparison of security features.

Security Feature	Chadwick et al. [16]	Quantin et al. [8]	Riedl et al. [6]	Gajanayake et al. [2]	Sun et al. [9]	Jo & Chung [17]	Seol et al. [18]	PAX
Mutual authentication								✓
Preserving anonymity		✓	✓		✓			✓
Pseudonym		✓	✓		✓			✓
Anti DoS	✓		✓		✓		✓	✓
Anti dataset attack							✓	✓
Anti MITM	✓	✓	✓		✓		✓	✓
Anti replay	✓		✓	✓	✓	✓		✓
Anti privileged insider					✓			✓
Anti traceability			✓		✓			✓
Anti impersonation								✓
Anti timing					✓			✓
Anti leakage			✓		✓			✓
Authorisation policies	✓			✓		✓	✓	✓

**Figure A2:**
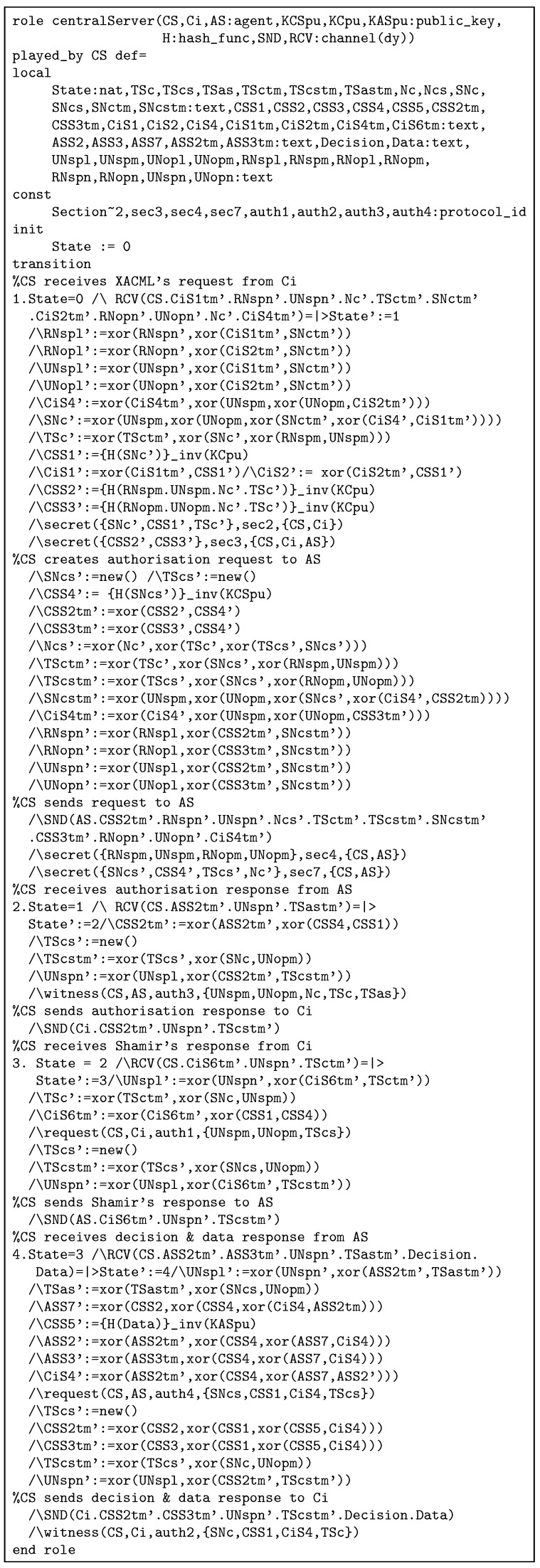
CS role of PAX in HLPSL.

**Figure A3:**
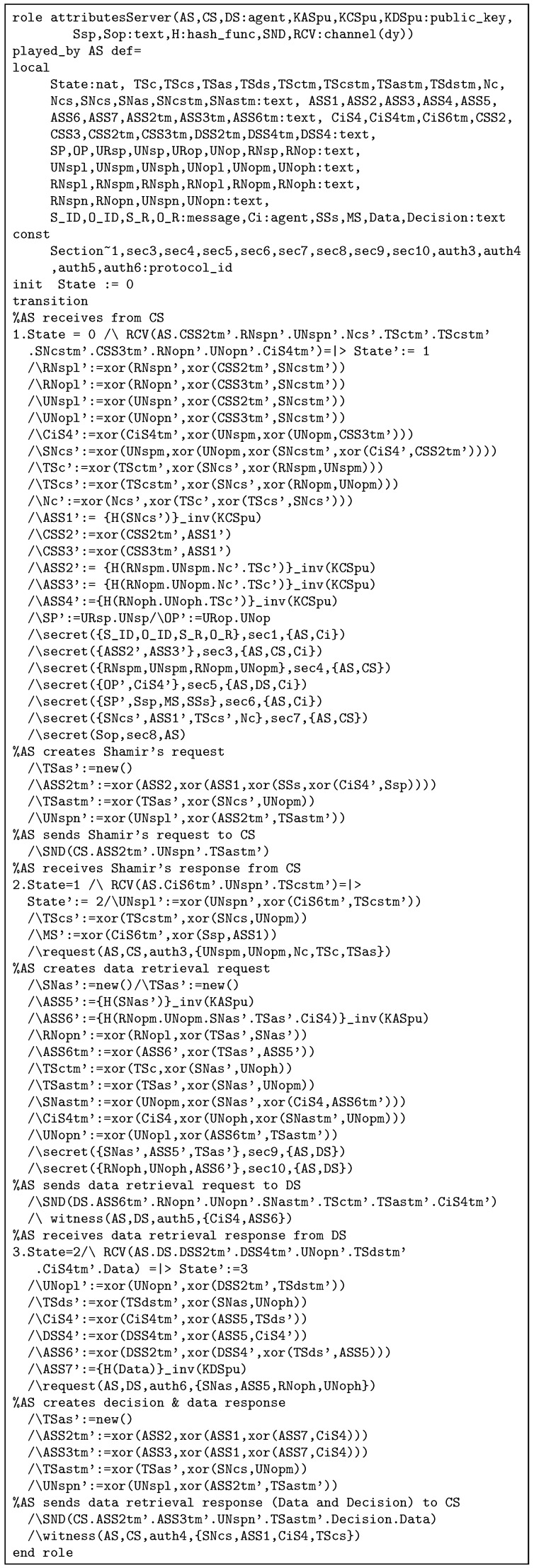
AS role of PAX in HLPSL.

## Abbreviations

The following abbreviations are used in this manuscript:

$C_{i}$	Client entity
CS, AS, DS	Central server, Attributes server and Data server
MS	Master secret/Master signature
SS	One secret sharing
$S_{ID}$, $O_{ID}$	Subject ID, Object ID
$S_{R}$, $O_{R}$	Subject role, Object role
$UR_{i}$	User’s role (patient, patient relative or provider)
$CN_{i}$	Client’s number
$RN_{i}$	Role’s number
$UN_{i}$	User’s number
SP, OP	Subject ’s pseudonym and Object ’s pseudonym
$S_{sp}$, $S_{op}$	Signature of SP and Signature of OP
$RN_{sp}$, $UN_{sp}$	$RN_{i}$, $UN_{i}$ for SP
$RN_{op}$, $UN_{op}$	$RN_{i}$, $UN_{i}$ for OP
*N*, SN	Random nonces and random secret nonce
TS	Timestamp
$C_{i}S_{j}$	Signature generated by $C_{i}$ and j is signature number
$CSS_{j}$	Signature generated by CS
$ASS_{j}$	Signature generated by AS
$DSS_{j}$	Signature generated by DS
||, ⊕, tm	Concatenation operation, Exclusive or operation and Temporary

## Appendix A

The following scripts represent roles in AVISPA tool:
